# Supplementary material for: CpG dinucleotide enrichment in the influenza A virus genome as a live attenuated vaccine development strategy
Source: PLoS Pathog. 2023 May 5;19(5):e1011357. doi: 10.1371/journal.ppat.1011357 (PMC10191365; doi:10.1371/journal.ppat.1011357)
Supplement: S1 Table — (DOCX) [file ppat.1011357.s001.docx]

**Table S1. Segment 1 modified PR8 sequences.**

>A/human/PuertoRico/8_seg1_wildtype

AGCGAAAGCAGGTCAATTATATTCAATATGGAAAGAATAAAAGAACTAAGAAATCTAATGTCGCAGTCTCGCACCCGCGAGATACTCACAAAAACCACCGTGGACCATATGGCCATAATCAAGAAGTACACATCAGGAAGACAGGAGAAGAACCCAGCACTTAGGATGAAATGGATGATGGCAATGAAATATCCAATTACAGCAGACAAGAGGATAACGGAAATGATTCCTGAGAGAAATGAGCAAGGACAAACTTTATGGAGTAAAATGAATGATGCCGGATCAGACCGAGTGATGGTATCACCACTGGCTGTGACATGGTGGAATAGGAATGGACCAATAACAAATACAGTTCATTATCCAAAAATCTACAAAACTTATTTTGAAAGAGTCGAAAGGCTAAAGCATGGAACCTTTGGCCCTGTCCATTTTAGAAACCAAGTCAAAATACGTCGGAGAGTTGACATAAATCCTGGTCATGCAGATCTCAGTGCCAAGGAGGCACAGGATGTAATCATGGAAGTTGTTTTCCCTAACGAAGTGGGAGCCAGGATACTAACATCGGAATCGCAACTAACGATAACCAAAGAGAAGAAAGAAGAACTCCAGGATTGCAAAATTTCTCCTTTGATGGTTGCATACATGTTGGAGAGAGAACTGGTCCGCAAAACGAGATTCCTCCCAGTGGCTGGTGGAACAAGCAGTGTGTACATTGAAGTGTTGCATTTGACTCAAGGAACATGCTGGGAACAGATGTATACTCCAGGAGGGGAAGTGAGGAATGATGATGTTGATCAAAGCTTGATTATTGCTGCTAGGAACATAGTGAGAAGAGCTGCAGTATCAGCAGATCCACTAGCATCTTTATTGGAGATGTGCCACAGCACACAGATTGGTGGAATTAGGATGGTAGACATCCTTAGGCAGAACCCAACAGAAGAGCAAGCCGTGGATATATGCAAGGCTGCAATGGGACTGAGAATTAGCTCATCCTTCAGTTTTGGTGGATTCACATTTAAGAGAACAAGCGGATCATCAGTCAAGAGAGAGGAAGAGGTGCTTACGGGCAATCTTCAAACATTGAAGATAAGAGTGCATGAGGGATATGAAGAGTTCACAATGGTTGGGAGAAGAGCAACAGCCATACTCAGAAAAGCAACCAGGAGATTGATTCAGCTGATAGTGAGTGGGAGAGACGAACAGTCGATTGCCGAAGCAATAATTGTGGCCATGGTATTTTCACAAGAGGATTGTATGATAAAAGCAGTCAGAGGTGATCTGAATTTCGTCAATAGGGCGAATCAGCGATTGAATCCTATGCATCAACTTTTAAGACATTTTCAGAAGGATGCGAAAGTGCTTTTTCAAAATTGGGGAGTTGAACCTATCGACAATGTGATGGGAATGATTGGGATATTGCCCGACATGACTCCAAGCATCGAGATGTCAATGAGAGGAGTGAGAATCAGCAAAATGGGTGTAGATGAGTACTCCAGCACGGAGAGGGTAGTGGTGAGCATTGACCGTTTTTTGAGAATCCGGGACCAACGAGGAAATGTACTACTGTCTCCCGAGGAGGTCAGTGAAACACAGGGAACAGAGAAACTGACAATAACTTACTCATCGTCAATGATGTGGGAGATTAATGGTCCTGAATCAGTGTTGGTCAATACCTATCAATGGATCATCAGAAACTGGGAAACTGTTAAAATTCAGTGGTCCCAGAACCCTACAATGCTATACAATAAAATGGAATTTGAACCATTTCAGTCTTTAGTACCTAAGGCCATTAGAGGCCAATACAGTGGGTTTGTAAGAACTCTGTTCCAACAAATGAGGGATGTGCTTGGGACATTTGATACCGCACAGATAATAAAACTTCTTCCCTTCGCAGCCGCTCCACCAAAGCAAAGTAGAATGCAGTTCTCCTCATTTACTGTGAATGTGAGGGGATCAGGAATGAGAATACTTGTAAGGGGCAATTCTCCTGTATTCAACTATAACAAGGCCACGAAGAGACTCACAGTTCTCGGAAAGGATGCTGGCACTTTAACTGAAGACCCAGATGAAGGCACAGCTGGAGTGGAGTCCGCTGTTCTGAGGGGATTCCTCATTCTGGGCAAAGAGAACAAGAGATATGGGCCAGCACTAAGCATCAATGAACTGAGCAACCTTGCGAAAGGAGAGAAGGCTAATGTGCTAATTGGGCAAGGAGACGTGGTGTTGGTAATGAAACGGAAACGGGACTCTAGCATACTTACTGACAGCCAGACAGCGACCAAAAGAATTCGGATGGCCATCAATTAGTGTCGAATAGTTTAAAAACGACCTTGTTTCTACT

> A/human/PuertoRico/8_seg1_CDLR

AGCGAAAGCAGGTCAATTATATTCAATATGGAAAGAATAAAAGAACTAAGAAATCTAATGTCGCAGTCTCGCACCCGCGAGATACTCACAAAAACCACCGTGGACCATATGGCCATAATCAAGAAGTACACATCAGGAAGACAGGAGAAGAACCCAGCACTTAGGATGAAATGGATGATGGCAATGAAATATCCAATTACAGCAGACAAGAGGATAACGGAAATGATTCCTGAGAGAAATGAGCAAGGACAAACTTTATGGAGTAAAATGAATGATGCCGGATCAGACCGAGTGATGGTATCACCACTGGCTGTGACATGGTGGAATAGGAATGGACCAATAACAAATACAGTTCATTATCCAAAAATCTACAAAACTTATTTTGAAAGAGTCGAAAGGCTAAAGCATGGAACCTTTGGCCCTGTCCATTTTAGAAACCAAGTCAAAATACGTCGGAGAGTTGACATAAATCCTGGTCATGCAGATCTCAGTGCCAAGGAGGCTCAGGATGTAATCATGGAAGTGGTGTTCCCAAACGAGGTTGGAGCAAGAATCCTCACTTCTGAGTCGCAACTAACAATAACTAAAGAGAAAAAAGAGGAGCTACAAGATTGCAAAATATCACCTTTGATGGTTGCATACATGTTGGAGAGAGAACTGGTCCGAAAGACGAGATTCCTACCCGTGGCCGGTGGGACCAGCAGTGTATACATAGAAGTGTTGCATTTGACTCAAGGGACGTGCTGGGAACAGATGTATACACCAGGGGGGGAAGTGAGAAATGATGATGTTGACCAGAGCTTGATAATTGCCGCGAGGAACATTGTGAGAAGAGCCGCCGTATCGGCAGATCCTCTAGCATCCTTATTGGAAATGTGTCACAGCACACAGATTGGTGGAATAAGAATGGTTGACATCCTTAGACAGAATCCCACAGAAGAGCAGGCTGTGGATATTTGCAAGGCTGCCATGGGGCTGAGAATCAGTTCATCATTTAGTTTTGGTGGATTCACTTTCAAGAGAACAAGCGGGTCATCAGTCAAGAGAGAGGAAGAAGTGCTAACGGGGAATCTCCAAACATTGAAAATAAGAGTTCATGAGGGCTATGAAGAGTTCACGATGGTTGGGAGGAGAGCAACCGCCATACTTAGAAAAGCAACGAGGAGATTGATACAACTGATAGTGAGTGGCAGAGACGAGCAATCTATTGCAGAAGCAATAATTGTGGCTATGGTGTTTTCCCAGGAAGATTGTATGATTAAAGCAGTCAGAGGAGACCTGAACTTTGTTAATAGGGCAAATCAACGATTGAACCCTATGCATCAACTTTTAAGACATTTTCAAAAGGATGCAAAAGTGCTTTTTCAAAATTGGGGAGTTGAACCAATCGACAATGTGATGGGCATGATAGGAATTTTGCCAGACATGACTCCAAGCATCGAGATGTCAATGAGAGGTGTCAGAATCAGCAAAATGGGTGTAGATGAATACTCGAGCACTGAGAGGGTAGTGGTCAGCATTGACCGATTTTTGAGAATTCGGGATCAACGAGGGAATGTTCTTCTGTCACCGGAAGAGGTGAGTGAAACTCAGGGAACTGAGAAACTGACAATAACATATTCCTCATCAATGATGTGGGAGATTAATGGCCCAGAATCAGTATTGGTGAATACATATCAATGGATCATCAGAAACTGGGAGACTGTTAAAATTCAGTGGTCTCAGAATCCCACTATGCTATACAATAAAATGGAGTTCGAGCCCTTCCAATCTTTAGTGCCTAAGGCCATTAGAGGCCAGTACAGTGGATTCGTAAGAACACTGTTCCAACAGATGAGAGATGTACTTGGGACATTTGATACAGCACAGATTATAAAACTGCTTCCATTTGCAGCCGCTCCACCAAAGCAAAGTAGAATGCAGTTCTCCTCATTTACTGTGAATGTGAGGGGATCAGGAATGAGAATACTTGTAAGGGGCAATTCTCCTGTATTCAACTATAACAAGGCCACGAAGAGACTCACAGTTCTCGGAAAGGATGCTGGCACTTTAACTGAAGACCCAGATGAAGGCACAGCTGGAGTGGAGTCCGCTGTTCTGAGGGGATTCCTCATTCTGGGCAAAGAGAACAAGAGATATGGGCCAGCACTAAGCATCAATGAACTGAGCAACCTTGCGAAAGGAGAGAAGGCTAATGTGCTAATTGGGCAAGGAGACGTGGTGTTGGTAATGAAACGGAAACGGGACTCTAGCATACTTACTGACAGCCAGACAGCGACCAAAAGAATTCGGATGGCCATCAATTAGTGTCGAATAGTTTAAAAACGACCTTGTTTCTACT

> A/human/PuertoRico/8_seg1_CpG-high

AGCGAAAGCAGGTCAATTATATTCAATATGGAAAGAATAAAAGAACTAAGAAATCTAATGTCGCAGTCTCGCACCCGCGAGATACTCACAAAAACCACCGTGGACCATATGGCCATAATCAAGAAGTACACATCAGGAAGACAGGAGAAGAACCCAGCACTTAGGATGAAATGGATGATGGCAATGAAATATCCAATTACAGCAGACAAGAGGATAACGGAAATGATTCCTGAGAGAAATGAGCAAGGACAAACTTTATGGAGTAAAATGAATGATGCCGGATCAGACCGAGTGATGGTATCACCACTGGCTGTGACATGGTGGAATAGGAATGGACCAATAACAAATACAGTTCATTATCCAAAAATCTACAAAACTTATTTTGAAAGAGTCGAAAGGCTAAAGCATGGAACCTTTGGCCCTGTCCATTTTAGAAACCAAGTCAAAATACGTCGGAGAGTTGACATAAATCCTGGTCATGCAGATCTCAGTGCAAAAGAAGCTCAAGATGTGATCATGGAAGTAGTTTTTCCGAATGAAGTCGGCGCGCGAATACTAACGTCGGAATCGCAACTGACGATAACGAAAGAGAAAAAAGAAGAACTTCAGGATTGCAAAATTTCGCCATTAATGGTCGCGTATATGTTGGAACGCGAACTCGTTCGAAAAACGCGATTTCTTCCGGTTGCCGGAGGAACGAGCAGCGTGTACATAGAAGTATTACATTTGACGCAAGGAACGTGTTGGGAACAAATGTATACGCCGGGCGGCGAAGTTCGAAACGATGACGTAGATCAAAGTTTGATTATAGCGGCTCGAAATATCGTGCGACGCGCGGCGGTTTCCGCCGATCCGCTCGCGTCATTATTGGAAATGTGTCACAGCACGCAGATAGGAGGAATCCGAATGGTCGACATTCTACGGCAAAATCCGACGGAAGAACAAGCAGTCGACATTTGCAAGGCCGCGATGGGACTGCGAATCAGTTCGTCTTTTAGTTTTGGCGGATTCACGTTCAAACGTACGAGTGGATCGTCAGTGAAGCGCGAAGAAGAAGTTCTGACGGGAAATCTTCAAACATTGAAAATCCGAGTGCATGAAGGATACGAAGAATTCACTATGGTCGGTCGACGCGCGACAGCGATACTACGAAAAGCGACGCGTCGATTAATTCAGCTGATAGTTAGCGGACGGGACGAACAGTCGATCGCCGAAGCGATCATCGTAGCGATGGTGTTTTCGCAAGAAGATTGCATGATAAAAGCCGTGCGAGGCGATCTAAATTTCGTAAATCGAGCGAATCAACGATTGAATCCGATGCATCAACTTTTGCGACATTTTCAGAAAGACGCGAAAGTGCTTTTTCAAAATTGGGGAGTCGAACCGATCGACAACGTAATGGGAATGATAGGTATTTTACCCGACATGACGCCGAGCATCGAAATGTCGATGCGCGGCGTTCGAATCAGCAAAATGGGCGTCGACGAATATTCAAGCACAGAGCGAGTCGTTGTAAGCATTGATCGTTTTTTACGAATACGCGATCAACGAGGTAACGTTCTTCTTTCGCCGGAAGAAGTAAGTGAAACGCAAGGTACGGAGAAACTTACGATCACGTATTCGTCGTCAATGATGTGGGAGATCAACGGTCCAGAATCCGTCTTGGTAAACACGTATCAATGGATCATCCGAAATTGGGAAACAGTGAAAATTCAGTGGTCGCAAAATCCGACGATGCTTTACAACAAAATGGAATTCGAACCGTTTCAATCATTAGTTCCAAAAGCGATTCGCGGTCAATATAGTGGATTTGTTCGAACGCTGTTTCAACAAATGCGTGACGTACTCGGAACGTTCGACACCGCGCAAATTATTAAACTTCTACCGTTCGCAGCCGCTCCACCAAAGCAAAGTAGAATGCAGTTCTCCTCATTTACTGTGAATGTGAGGGGATCAGGAATGAGAATACTTGTAAGGGGCAATTCTCCTGTATTCAACTATAACAAGGCCACGAAGAGACTCACAGTTCTCGGAAAGGATGCTGGCACTTTAACTGAAGACCCAGATGAAGGCACAGCTGGAGTGGAGTCCGCTGTTCTGAGGGGATTCCTCATTCTGGGCAAAGAGAACAAGAGATATGGGCCAGCACTAAGCATCAATGAACTGAGCAACCTTGCGAAAGGAGAGAAGGCTAATGTGCTAATTGGGCAAGGAGACGTGGTGTTGGTAATGAAACGGAAACGGGACTCTAGCATACTTACTGACAGCCAGACAGCGACCAAAAGAATTCGGATGGCCATCAATTAGTGTCGAATAGTTTAAAAACGACCTTGTTTCTACT

> A/human/PuertoRico/8_seg1_5’-CpGH

AGCGAAAGCAGGTCAATTATATTCAATATGGAAAGAATAAAAGAACTAAGAAATCTAATGTCGCAGTCTCGCACCCGCGAGATACTCACAAAAACCACCGTGGACCATATGGCCATAATCAAGAAGTACACATCAGGAAGACAGGAGAAGAACCCAGCACTTAGGATGAAATGGATGATGGCAATGAAATATCCAATTACAGCAGACAAGAGGATAACGGAAATGATTCCTGAGAGAAATGAGCAAGGACAAACTTTATGGAGTAAAATGAATGATGCCGGATCAGACCGAGTGATGGTATCACCACTGGCTGTGACATGGTGGAATAGGAATGGACCAATAACAAATACAGTTCATTATCCAAAAATCTACAAAACTTATTTTGAAAGAGTCGAAAGGCTAAAGCATGGAACCTTTGGCCCTGTCCATTTTAGAAACCAAGTCAAAATACGTCGGAGAGTTGACATAAATCCTGGTCATGCAGATCTCAGTGCAAAAGAAGCTCAAGATGTGATCATGGAAGTAGTTTTTCCGAATGAAGTCGGCGCGCGAATACTAACGTCGGAATCGCAACTGACGATAACGAAAGAGAAAAAAGAAGAACTTCAGGATTGCAAAATTTCGCCATTAATGGTCGCGTATATGTTGGAACGCGAACTCGTTCGAAAAACGCGATTTCTTCCGGTTGCCGGAGGAACGAGCAGCGTGTACATAGAAGTATTACATTTGACGCAAGGAACGTGTTGGGAACAAATGTATACGCCGGGCGGCGAAGTTCGAAACGATGACGTAGATCAAAGTTTGATTATAGCGGCTCGAAATATCGTGCGACGCGCGGCGGTTTCCGCCGATCCGCTCGCGTCATTATTGGAAATGTGTCACAGCACGCAGATAGGAGGAATCCGAATGGTCGACATTCTACGGCAAAATCCGACGGAAGAACAAGCAGTCGACATTTGCAAGGCCGCGATGGGACTGCGAATCAGTTCGTCTTTTAGTTTTGGCGGATTCACGTTCAAACGTACGAGTGGATCGTCAGTGAAGCGCGAAGAAGAAGTTCTGACGGGAAATCTTCAAACATTGAAAATCCGAGTGCATGAAGGATACGAAGAATTCACTATGGTCGGTCGACGCGCGACAGCGATACTACGAAAAGCGACGCGTCGATTAATTCAGCTGATAGTTAGCGGACGGGACGAACAGTCGATCGCCGAAGCGATCATCGTAGCGATGGTGTTTTCGCAAGAAGATTGCATGATAAAAGCCGTGCGAGGCGATCTAAATTTCGTAAATCGAGCGAATCAACGATTGAATCCGATGCATCAACTTTTAAGACATTTTCAGAAGGATGCGAAAGTGCTTTTTCAAAATTGGGGAGTTGAACCTATCGACAATGTGATGGGAATGATTGGGATATTGCCCGACATGACTCCAAGCATCGAGATGTCAATGAGAGGAGTGAGAATCAGCAAAATGGGTGTAGATGAGTACTCCAGCACGGAGAGGGTAGTGGTGAGCATTGACCGTTTTTTGAGAATCCGGGACCAACGAGGAAATGTACTACTGTCTCCCGAGGAGGTCAGTGAAACACAGGGAACAGAGAAACTGACAATAACTTACTCATCGTCAATGATGTGGGAGATTAATGGTCCTGAATCAGTGTTGGTCAATACCTATCAATGGATCATCAGAAACTGGGAAACTGTTAAAATTCAGTGGTCCCAGAACCCTACAATGCTATACAATAAAATGGAATTTGAACCATTTCAGTCTTTAGTACCTAAGGCCATTAGAGGCCAATACAGTGGGTTTGTAAGAACTCTGTTCCAACAAATGAGGGATGTGCTTGGGACATTTGATACCGCACAGATAATAAAACTTCTTCCCTTCGCAGCCGCTCCACCAAAGCAAAGTAGAATGCAGTTCTCCTCATTTACTGTGAATGTGAGGGGATCAGGAATGAGAATACTTGTAAGGGGCAATTCTCCTGTATTCAACTATAACAAGGCCACGAAGAGACTCACAGTTCTCGGAAAGGATGCTGGCACTTTAACTGAAGACCCAGATGAAGGCACAGCTGGAGTGGAGTCCGCTGTTCTGAGGGGATTCCTCATTCTGGGCAAAGAGAACAAGAGATATGGGCCAGCACTAAGCATCAATGAACTGAGCAACCTTGCGAAAGGAGAGAAGGCTAATGTGCTAATTGGGCAAGGAGACGTGGTGTTGGTAATGAAACGGAAACGGGACTCTAGCATACTTACTGACAGCCAGACAGCGACCAAAAGAATTCGGATGGCCATCAATTAGTGTCGAATAGTTTAAAAACGACCTTGTTTCTACT

> A/human/PuertoRico/8_seg1_3’-CpGH

AGCGAAAGCAGGTCAATTATATTCAATATGGAAAGAATAAAAGAACTAAGAAATCTAATGTCGCAGTCTCGCACCCGCGAGATACTCACAAAAACCACCGTGGACCATATGGCCATAATCAAGAAGTACACATCAGGAAGACAGGAGAAGAACCCAGCACTTAGGATGAAATGGATGATGGCAATGAAATATCCAATTACAGCAGACAAGAGGATAACGGAAATGATTCCTGAGAGAAATGAGCAAGGACAAACTTTATGGAGTAAAATGAATGATGCCGGATCAGACCGAGTGATGGTATCACCACTGGCTGTGACATGGTGGAATAGGAATGGACCAATAACAAATACAGTTCATTATCCAAAAATCTACAAAACTTATTTTGAAAGAGTCGAAAGGCTAAAGCATGGAACCTTTGGCCCTGTCCATTTTAGAAACCAAGTCAAAATACGTCGGAGAGTTGACATAAATCCTGGTCATGCAGATCTCAGTGCCAAGGAGGCACAGGATGTAATCATGGAAGTTGTTTTCCCTAACGAAGTGGGAGCCAGGATACTAACATCGGAATCGCAACTAACGATAACCAAAGAGAAGAAAGAAGAACTCCAGGATTGCAAAATTTCTCCTTTGATGGTTGCATACATGTTGGAGAGAGAACTGGTCCGCAAAACGAGATTCCTCCCAGTGGCTGGTGGAACAAGCAGTGTGTACATTGAAGTGTTGCATTTGACTCAAGGAACATGCTGGGAACAGATGTATACTCCAGGAGGGGAAGTGAGGAATGATGATGTTGATCAAAGCTTGATTATTGCTGCTAGGAACATAGTGAGAAGAGCTGCAGTATCAGCAGATCCACTAGCATCTTTATTGGAGATGTGCCACAGCACACAGATTGGTGGAATTAGGATGGTAGACATCCTTAGGCAGAACCCAACAGAAGAGCAAGCCGTGGATATATGCAAGGCTGCAATGGGACTGAGAATTAGCTCATCCTTCAGTTTTGGTGGATTCACATTTAAGAGAACAAGCGGATCATCAGTCAAGAGAGAGGAAGAGGTGCTTACGGGCAATCTTCAAACATTGAAGATAAGAGTGCATGAGGGATATGAAGAGTTCACAATGGTTGGGAGAAGAGCAACAGCCATACTCAGAAAAGCAACCAGGAGATTGATTCAGCTGATAGTGAGTGGGAGAGACGAACAGTCGATTGCCGAAGCAATAATTGTGGCCATGGTATTTTCACAAGAGGATTGTATGATAAAAGCAGTCAGAGGTGATCTGAATTTCGTCAATAGGGCGAATCAGCGATTGAATCCTATGCATCAACTTTTGCGACATTTTCAGAAAGACGCGAAAGTGCTTTTTCAAAATTGGGGAGTCGAACCGATCGACAACGTAATGGGAATGATAGGTATTTTACCCGACATGACGCCGAGCATCGAAATGTCGATGCGCGGCGTTCGAATCAGCAAAATGGGCGTCGACGAATATTCAAGCACAGAGCGAGTCGTTGTAAGCATTGATCGTTTTTTACGAATACGCGATCAACGAGGTAACGTTCTTCTTTCGCCGGAAGAAGTAAGTGAAACGCAAGGTACGGAGAAACTTACGATCACGTATTCGTCGTCAATGATGTGGGAGATCAACGGTCCAGAATCCGTCTTGGTAAACACGTATCAATGGATCATCCGAAATTGGGAAACAGTGAAAATTCAGTGGTCGCAAAATCCGACGATGCTTTACAACAAAATGGAATTCGAACCGTTTCAATCATTAGTTCCAAAAGCGATTCGCGGTCAATATAGTGGATTTGTTCGAACGCTGTTTCAACAAATGCGTGACGTACTCGGAACGTTCGACACCGCGCAAATTATTAAACTTCTACCGTTCGCAGCCGCTCCACCAAAGCAAAGTAGAATGCAGTTCTCCTCATTTACTGTGAATGTGAGGGGATCAGGAATGAGAATACTTGTAAGGGGCAATTCTCCTGTATTCAACTATAACAAGGCCACGAAGAGACTCACAGTTCTCGGAAAGGATGCTGGCACTTTAACTGAAGACCCAGATGAAGGCACAGCTGGAGTGGAGTCCGCTGTTCTGAGGGGATTCCTCATTCTGGGCAAAGAGAACAAGAGATATGGGCCAGCACTAAGCATCAATGAACTGAGCAACCTTGCGAAAGGAGAGAAGGCTAATGTGCTAATTGGGCAAGGAGACGTGGTGTTGGTAATGAAACGGAAACGGGACTCTAGCATACTTACTGACAGCCAGACAGCGACCAAAAGAATTCGGATGGCCATCAATTAGTGTCGAATAGTTTAAAAACGACCTTGTTTCTACT

> A/human/PuertoRico/8_seg1_CpGM

AGCGAAAGCAGGTCAATTATATTCAATATGGAAAGAATAAAAGAACTAAGAAATCTAATGTCGCAGTCTCGCACCCGCGAGATACTCACAAAAACCACCGTGGACCATATGGCCATAATCAAGAAGTACACATCAGGAAGACAGGAGAAGAACCCAGCACTTAGGATGAAATGGATGATGGCAATGAAATATCCAATTACAGCAGACAAGAGGATAACGGAAATGATTCCTGAGAGAAATGAGCAAGGACAAACTTTATGGAGTAAAATGAATGATGCCGGATCAGACCGAGTGATGGTATCACCACTGGCTGTGACATGGTGGAATAGGAATGGACCAATAACAAATACAGTTCATTATCCAAAAATCTACAAAACTTATTTTGAAAGAGTCGAAAGGCTAAAGCATGGAACCTTTGGCCCTGTCCATTTTAGAAACCAAGTCAAAATACGTCGGAGAGTTGACATAAATCCTGGTCATGCAGATCTCAGTGCCAAGGAGGCGCAGGATGTAATCATGGAAGTTGTTTTCCCGAACGAAGTGGGAGCCCGGATACTAACATCTGAATCCCAACTAACGATAACGAAAGAGAAGAAAGAAGAACTCCAGGATTGCAAAATTTCGCCTTTGATGGTTGCGTACATGTTGGAGAGAGAACTCGTCCGCAAAACTCGATTCCTCCCAGTGGCTGGCGGAACAAGCAGCGTGTACATTGAAGTGTTGCATTTGACGCAAGGAACATGCTGGGAACAGATGTATACGCCAGGAGGGGAAGTGAGGAATGATGACGTTGATCAAAGCTTGATTATTGCTGCTCGGAACATAGTGAGAAGAGCTGCGGTATCAGCAGATCCACTAGCGTCTTTATTGGAGATGTGCCACAGCACGCAGATTGGTGGAATTAGGATGGTCGACATCCTTAGGCAGAACCCAACGGAAGAGCAAGCTGTCGATATATGCAAGGCTGCGATGGGACTGAGAATTAGCTCGTCCTTCAGTTTTGGCGGATTCACATTTAAGAGAACAAGCGGATCATCAGTCAAGCGAGAGGAAGAGGTGCTTACGGGCAATCTTCAAACATTGAAGATACGAGTGCATGAGGGATACGAAGAGTTCACAATGGTTGGGAGAAGAGCGACAGCCATACTCCGAAAAGCAACCAGGCGATTGATTCAGCTGATAGTGAGCGGGAGAGACGAACAGTCGATTGCCGAAGCGATAATTGTGGCCATGGTATTTTCGCAAGAGGATTGTATGATAAAAGCAGTCCGAGGTGATCTGAATTTCGTCAATAGGGCCAATCAGCGATTGAATCCTATGCATCAACTTTTACGACATTTTCAGAAGGATGCGAAAGTGCTTTTTCAAAATTGGGGAGTCGAACCTATTGACAACGTGATGGGAATGATTGGGATATTGCCCGACATGACTCCAAGCATTGAGATGTCGATGAGAGGAGTGCGAATCAGCAAAATGGGTGTAGACGAGTACTCCAGCACGGAGAGGGTCGTGGTGAGCATTGACCGTTTTTTGCGAATCAGGGACCAAAGGGGAAACGTACTACTGTCTCCCGAGGAGGTCAGTGAAACGCAGGGAACAGAGAAACTGACAATAACGTACTCATCGTCAATGATGTGGGAGATTAACGGTCCTGAATCAGTGTTGGTCAATACGTATCAATGGATCATCCGAAACTGGGAAACCGTTAAAATTCAGTGGTCCCAGAACCCTACGATGCTATACAATAAAATGGAATTCGAACCGTTTCAGTCTTTAGTACCTAAGGCCATTCGAGGCCAATACAGTGGGTTTGTAAGAACGCTGTTCCAACAAATGAGGGACGTGCTCGGGACATTTGATACTGCGCAGATAATAAAACTTCTTCCCTTCGCAGCTGCTCCACCGAAGCAAAGTAGAATGCAGTTCTCCTCATTTACTGTGAATGTGAGGGGATCAGGAATGAGAATACTTGTAAGGGGCAATTCTCCTGTATTCAACTATAACAAGGCCACGAAGAGACTCACAGTTCTCGGAAAGGATGCTGGCACTTTAACTGAAGACCCAGATGAAGGCACAGCTGGAGTGGAGTCCGCTGTTCTGAGGGGATTCCTCATTCTGGGCAAAGAGAACAAGAGATATGGGCCAGCACTAAGCATCAATGAACTGAGCAACCTTGCGAAAGGAGAGAAGGCTAATGTGCTAATTGGGCAAGGAGACGTGGTGTTGGTAATGAAACGGAAACGGGACTCTAGCATACTTACTGACAGCCAGACAGCGACCAAAAGAATTCGGATGGCCATCAATTAGTGTCGAATAGTTTAAAAACGACCTTGTTTCTACT

> A/human/PuertoRico/8_seg1_UpAH

AGCGAAAGCAGGTCAATTATATTCAATATGGAAAGAATAAAAGAACTAAGAAATCTAATGTCGCAGTCTCGCACCCGCGAGATACTCACAAAAACCACCGTGGACCATATGGCCATAATCAAGAAGTACACATCAGGAAGACAGGAGAAGAACCCAGCACTTAGGATGAAATGGATGATGGCAATGAAATATCCAATTACAGCAGACAAGAGGATAACGGAAATGATTCCTGAGAGAAATGAGCAAGGACAAACTTTATGGAGTAAAATGAATGATGCCGGATCAGACCGAGTGATGGTATCACCACTGGCTGTGACATGGTGGAATAGGAATGGACCAATAACAAATACAGTTCATTATCCAAAAATCTACAAAACTTATTTTGAAAGAGTCGAAAGGCTAAAGCATGGAACCTTTGGCCCTGTCCATTTTAGAAACCAAGTCAAAATACGTCGGAGAGTTGACATAAATCCTGGTCATGCAGATCTCAGTGCTAAGGAGGCCCAGGACGTAATTATGGAGGTAGTATTCCCTAACGAGGTAGGAGCTAGGATACTAACCTCTGAGTCCCAGCTAACTATAACTAAAGAGAAGAAAGAGGAACTCCAGGACTGTAAGATATCGCCTTTAATGGTAGCATATATGTTAGAGAGGGAACTAGTAAGGAAGACTAGGTTCCTACCCGTAGCAGGGGGTACTAGTAGTGTATATATAGAGGTATTACACTTAACCCAAGGTACCTGCTGGGAGCAGATGTATACTCCAGGAGGGGAGGTAAGGAACGACGACGTAGACCAAAGCTTAATTATAGCTGCTAGGAACATTGTAAGAAGAGCAGCAGTATCCGCAGATCCCCTAGCCTCATTATTAGAGATGTGCCATAGTACACAGATAGGGGGTATTAGGATGGTAGATATTCTAAGGCAGAACCCTACAGAGGAGCAAGCAGTCGATATATGTAAGGCCGCTATGGGTCTTAGGATAAGCTCATCCTTTAGTTTTGGGGGCTTTACTTTTAAGAGGACTAGCGGCTCATCCGTAAAGAGAGAGGAAGAGGTACTTACAGGTAACCTACAGACATTAAAGATAAGGGTACATGAGGGATACGAGGAGTTTACTATGGTAGGTAGAAGGGCTACAGCAATACTAAGAAAAGCTACTAGGAGGTTAATACAGCTAATAGTAAGTGGTAGGGATGAACAGTCTATAGCAGAAGCCATAATAGTTGCTATGGTCTTTTCACAGGAGGACTGTATGATAAAAGCCGTTAGGGGGGACCTTAACTTTGTAAATAGGGCTAACCAGAGGTTGAACCCTATGCACCAACTATTAAGGCACTTCCAGAAGGACGCTAAAGTACTATTCCAAAACTGGGGGGTAGAACCTATAGATAACGTAATGGGTATGATTGGTATATTACCTGATATGACACCTAGCATAGAGATGTCTATGAGAGGGGTAAGAATAAGTAAGATGGGAGTGGACGAGTACTCTAGCACTGAGAGGGTAGTAGTAAGTATCGATAGGTTCTTAAGAATAAGGGACCAGAGGGGAAATGTACTACTATCCCCCGAGGAGGTAAGCGAAACCCAGGGTACAGAGAAACTTACTATAACATACTCATCCTCTATGATGTGGGAGATAAACGGGCCTGAGTCAGTTTTAGTAAATACCTACCAATGGATTATTAGAAACTGGGAAACAGTTAAGATACAGTGGTCCCAGAACCCTACTATGCTATATAACAAGATGGAGTTCGAACCCTTCCAGTCTTTAGTACCTAAGGCTATCAGAGGCCAGTATAGTGGGTTTGTAAGGACCCTATTCCAGCAGATGAGGGATGTACTAGGTACCTTTGATACTGCACAGATAATAAAGCTACTACCCTTCGCGGCTGCTCCCCCTAAGCAGAGCAGAATGCAGTTCTCCTCATTTACTGTGAATGTGAGGGGATCAGGAATGAGAATACTTGTAAGGGGCAATTCTCCTGTATTCAACTATAACAAGGCCACGAAGAGACTCACAGTTCTCGGAAAGGATGCTGGCACTTTAACTGAAGACCCAGATGAAGGCACAGCTGGAGTGGAGTCCGCTGTTCTGAGGGGATTCCTCATTCTGGGCAAAGAGAACAAGAGATATGGGCCAGCACTAAGCATCAATGAACTGAGCAACCTTGCGAAAGGAGAGAAGGCTAATGTGCTAATTGGGCAAGGAGACGTGGTGTTGGTAATGAAACGGAAACGGGACTCTAGCATACTTACTGACAGCCAGACAGCGACCAAAAGAATTCGGATGGCCATCAATTAGTGTCGAATAGTTTAAAAACGACCTTGTTTCTACT
