# Supplementary material for: CpG dinucleotide enrichment in the influenza A virus genome as a live attenuated vaccine development strategy
Source: PLoS Pathog. 2023 May 5;19(5):e1011357. doi: 10.1371/journal.ppat.1011357 (PMC10191365; doi:10.1371/journal.ppat.1011357)
Supplement: S2 Table — S2A Table. Primers used for site directed mutagenesis of IAV PR8 segment 8. S2B Table. Primers for amplification of influenza A virus A/Puerto Rico/8/1934 (PR8) viral transcripts by qPCR. S2C Table. Primers for amplification of specific bisulphite converted RNA regions. S2D Table. Primers for generation of in vitro transcription amplicon templates. S2E Table. Primers for Northern blotting probe amplicon templates. S2F Table. Primers for overlapping full genome sequencing. (DOCX) [file ppat.1011357.s002.docx]

**Table S2. Primers used for mutagenesis and sequencing.**

**Table S2A.** Primers used for site directed mutagenesis of IAV PR8 segment 8.

| **Target** | **F primer** | **R primer** |
| --- | --- | --- |
| Seg8 L95S99A | TGACATGACTGCTGAGGAAATGGCAAGG | CCTTGCCATTTCCTCAGCAGTCATGTCA |
| Seg8 E9697A | ATGACTCTTGCGGCAATGTCAAGGG | CCCTTGACATTGCCGCAAGAGTCAT |
| Seg8 P107A | CATGCTCATAGCCAAGCAGAAAG | CTTTCTGCTTGGCTATGAGCATG |
| Seg8 P107K108A | GCTCATAGCCGCGCAGAAAGTGG | CCACTTTCTGCGCGGCTATGAGC |
| Seg8 P107K108Q109A | CATAGCCGCGGCGAAAGTGGCAG | CTGCCACTTTCGCCGCGGCTATG |
| Seg8 P107K108Q109K110A | AGCCGCGGCGGCAGTGGCAGGC | GCCTGCCACTGCCGCCGCGGCT |

**Table S2B.** Primers for amplification of influenza A virus A/Puerto Rico/8/1934 (PR8) viral transcripts by qPCR.

| **Target** | **Forward primer** | **Reverse primer** |
| --- | --- | --- |
| PR8 segment 1 | GGCTAATGTGCTAATTGGGCAAG | GGCTGTCAGTAAGTATGCTAGAG |
| PR8 segment 5 | ATCATGGCGTCTCAAGGCACCAA | GCTCTGATTTCAGTGGCATTCTGG |

**Table S2C.** Primers for amplification of specific bisulphite converted RNA regions.

| **Target** | **Forward primer** | **Reverse primer** |
| --- | --- | --- |
| PR8 106-304 | CATATAACCATAATCAAAAAATACACATC | GTGATATTATTATTTGGTTTGATTTGG |
| Chicken rRNA 3163-3307 | GTGATTTTTGTTTAGTGTTTTGAATG | AAATCTCATTCATCCATTCATACAC |

**Table S2D.** Primers for generation of *in vitro* transcription amplicon templates.

| **Target** | **Forward primer** | **Reverse primer** |
| --- | --- | --- |
| T7 Seg1 -sense | AGCGAAAGCAGGTCAATTATATTC | TAATACGACTCACTATAGGGAGTAGAAACAAGGTCGTTTTTAAACTA |
| T7 Seg1 +sense | TAATACGACTCACTATAGGGAGCGAAAGCAGGTCAATTATATTC | AGTAGAAACAAGGTCGTTTTTAAACTA |
| T7 Seg5 -sense | AGCAAAAGCAGGGTAGATAATCA | TAATACGACTCACTATAGGGAGTAGAAACAAGGGTATTTTTCTTTAATTGT |
| T7 Seg5 +sense | TAATACGACTCACTATAGGGAGCAAAAGCAGGGTAGATAATCA | AGTAGAAACAAGGGTATTTTTCTTTAATTGT |

**Table S2E.** Primers for Northern blotting probe amplicon templates.

| **Target** | **Forward primer** | **Reverse primer** |
| --- | --- | --- |
| PR8 segment 1 -sense probe | TAATACGACTCACTATAGGGGCCGCTCCACCAAAGCAAAG | TTCGACACTAATTGATGGCC |
| PR8 segment 1 +sense probe | GCCGCTCCACCAAAGCAAAG | TAATACGACTCACTATAGGGTTCGACACTAATTGATGGCC |
| PR8 segment 5 -sense probe | TAATACGACTCACTATAGGGCAAAAGCAGGGTAGATAATC | TCACCATTATTAGCTTGGCG |
| PR8 segment 5 +sense probe | CAAAAGCAGGGTAGATAATC | TAATACGACTCACTATAGGGTCACCATTATTAGCTTGGCG |

**Table S2F.** Primers for overlapping full genome sequencing.

| **Target** | **Forward primer** | **Reverse primer** |
| --- | --- | --- |
| Seg1 amplicon A | AGCGAAAGCAGGTCAATTAT | CCATGCTTTAGCCTTTCGAC |
| Seg1 amplicon B | ACATGGTGGAATAGGAATGG | TGCAACACTTCAATGTACAC |
| Seg1 amplicon C | CAAAACGAGATTCCTCCCAG | TGAAGATTGCCCGTAAGCAC |
| Seg1 amplicon D | AAGGCTGCAATGGGACTGAG | AGCACTTTCGCATCCTTCTG |
| Seg1 amplicon E | GCGATTGAATCCTATGCATC | TTGTAGGGTTCTGGGACCAC |
| Seg1 amplicon F | ATGGATCATCAGAAACTGGG | GGACTCCACTCCAGCTGTGC |
| Seg1 amplicon G | GCCGCTCCACCAAAGCAAAG | TTCGACACTAATTGATGGCC |
| Seg2 amplicon A | GAAAGCAGGCAAACCATTTG | TCATAGGTCTGTCGGCCTTG |
| Seg2 amplicon B | AACGATGGAGGTTGTTCAGC | CCCTGGGGTTGCAATTGCTC |
| Seg2 amplicon C | CAATGACCAAAGATGCTGAG | CTTGCTCTCAAACATATACCC |
| Seg2 amplicon D | AATCAGCCCGAATGGTTCAG | GTCGACTCCGGCTTGAATCC |
| Seg2 amplicon E | TGGTCTTCAATCCTCTGACG | GGGTTTGCTCCCACAGTTTC |
| Seg2 amplicon F | ATGCCATAGAGGTGACACAC | AATTGCAGCACCTTTGGTAC |
| Seg2 amplicon G | CCCATTTGTCAGCCATAAAG | GAAGGACAAGCTAAATTCAC |
| Seg3 amplicon A | AGCGAAAGCAGGTACTGATC | ACTTCTCTCCTTGTTACTCC |
| Seg3 amplicon B | GGCTGAGAAACCAAAGTTTC | GAATCCATCCACATAGGCTC |
| Seg3 amplicon C | TTGCCGACCAAAGTCTCCCG | TCATTCTCAATGTCCTGCAG |
| Seg3 amplicon D | GTTAAACCACACGAAAAGGG | ATGTATTCTGTGGCTCTGCAG |
| Seg3 amplicon E | ACATTGCAAGCATGAGAAGG | GAGGTTCCATTTGTTCTCAC |
| Seg3 amplicon F | AAGAAGTGCCATAGGCCAGG | CTCAATTGCTTCATATAGCC |
| Seg3 amplicon G | ATGATTGAAGCTGAGTCCTC | TTTTGGACAGTATGGATAGC |
| Seg4 amplicon A | CAACCAAAATGAAGGCAAAC | GGCCATGAGCTTTCTTTGGG |
| Seg4 amplicon B | GAGCTCAGTGTCATCATTCG | AATATTATTGTGTCTCCGGG |
| Seg4 amplicon C | TAAGAGATCAAGCTGGGAGG | AGCCTGATCCCTGTTCATTC |
| Seg4 amplicon D | ACTGGAATGATAGATGGATG | CATTTCTTACACTTTCCATGC |
| Seg4 amplicon E | GGATTTCTGGACATTTGGAC | ATTCTGCACTGCAAAGATCC |
| Seg5 amplicon A | CAAAAGCAGGGTAGATAATC | TCACCATTATTAGCTTGGCG |
| Seg5 amplicon B | GAGAGAACTCATCCTTTATG | TTCCGGCTCTCTCTCACTTG |
| Seg5 amplicon C | GAAATTTCAAACTGCTGCAC | TTGGAAGCAATTTGAACTCC |
| Seg5 amplicon D | CCAAGAGGGAAGCTTTCCAC | GTACTCCTCTGCATTGTCTC |
| Seg6 amplicon A | TGGATCAATCTGTCTGGTAG | ACAGGGCAGCTCATTAAGGCC |
| Seg6 amplicon B | TTCAAGTGGGACTGTTAAGG | GGTATCAGGGTAACAGGAAC |
| Seg6 amplicon C | GAGTTGAATGCACCTAATTC | CCGCTATACCCTGACCAATC |
| Seg6 amplicon D | GTGTATGTTGATGGAGCAAAC | GAACAGACTACTTGTCAATG |
| Seg7 amplicon A | GATGAGTCTTCTAACCGAGG | TGCCACTTCAGTGGTCACAG |
| Seg7 amplicon B | CCAAATAACATGGACAAAGC | GCTAGGATGAGTTCCAATGG |
| Seg7 amplicon C | GCTATGGAGCAAATGGCTGG | TTTTTACTCCAGCTCTATGC |
| Seg8 amplicon A | CATAATGGATCCAAACACTG | TCTCCAGCCGGTCAAAAATC |
| Seg8 amplicon B | AGAAGAATCCGATGAGGCAC | TTACTGCTTCTCCAAGCGAATC |
| Seg8 amplicon C | GACTCTAATATTGCTAAGGG | AAGCTGAAACGAGAAAGTTC |
|  |  |  |
| Seg1 CDLR amplicon B | ACATGGTGGAATAGGAATGG | TGCAACACTTCTATGTATAC |
| Seg1 CDLR amplicon C | AAAGACGAGATTCCTACCCG | TGGAGATTCCCCGTTAGCAC |
| Seg1 CDLR amplicon D | AAGGCTGCCATGGGGCTGAG | AGCACTTTTGCATCCTTTTG |
| Seg1 CDLR amplicon E | ACGATTGAACCCTATGCATC | TAGTGGGATTCTGAGACCAC |
| Seg1 CDLR amplicon F | ATGGATCATCAGAAACTGGG | GGACTCCACTCCAGCTGTGC |
|  |  |  |
| Seg1 CpGH amplicon B | ACATGGTGGAATAGGAATGG | TAATACTTCTATGTACACGC |
| Seg1 CpGH amplicon C | AAAAACGCGATTTCTTCCGG | TGAAGATTTCCCGTCAGAAC |
| Seg1 CpGH amplicon D | AAGGCCGCGATGGGACTGCG | AGCACTTTCGCGTCTTTCTG |
| Seg1 CpGH amplicon E | ACGATTGAATCCGATGCATC | TCGTCGGATTTTGCGACCAC |
| Seg1 CpGH amplicon F | ATGGATCATCCGAAATTGGG | GGACTCCACTCCAGCTGTGC |
