# Supplementary figures and images for: CpG dinucleotide enrichment in the influenza A virus genome as a live attenuated vaccine development strategy
Source: PLoS Pathog. 2023 May 5;19(5):e1011357. doi: 10.1371/journal.ppat.1011357 (PMC10191365; doi:10.1371/journal.ppat.1011357)

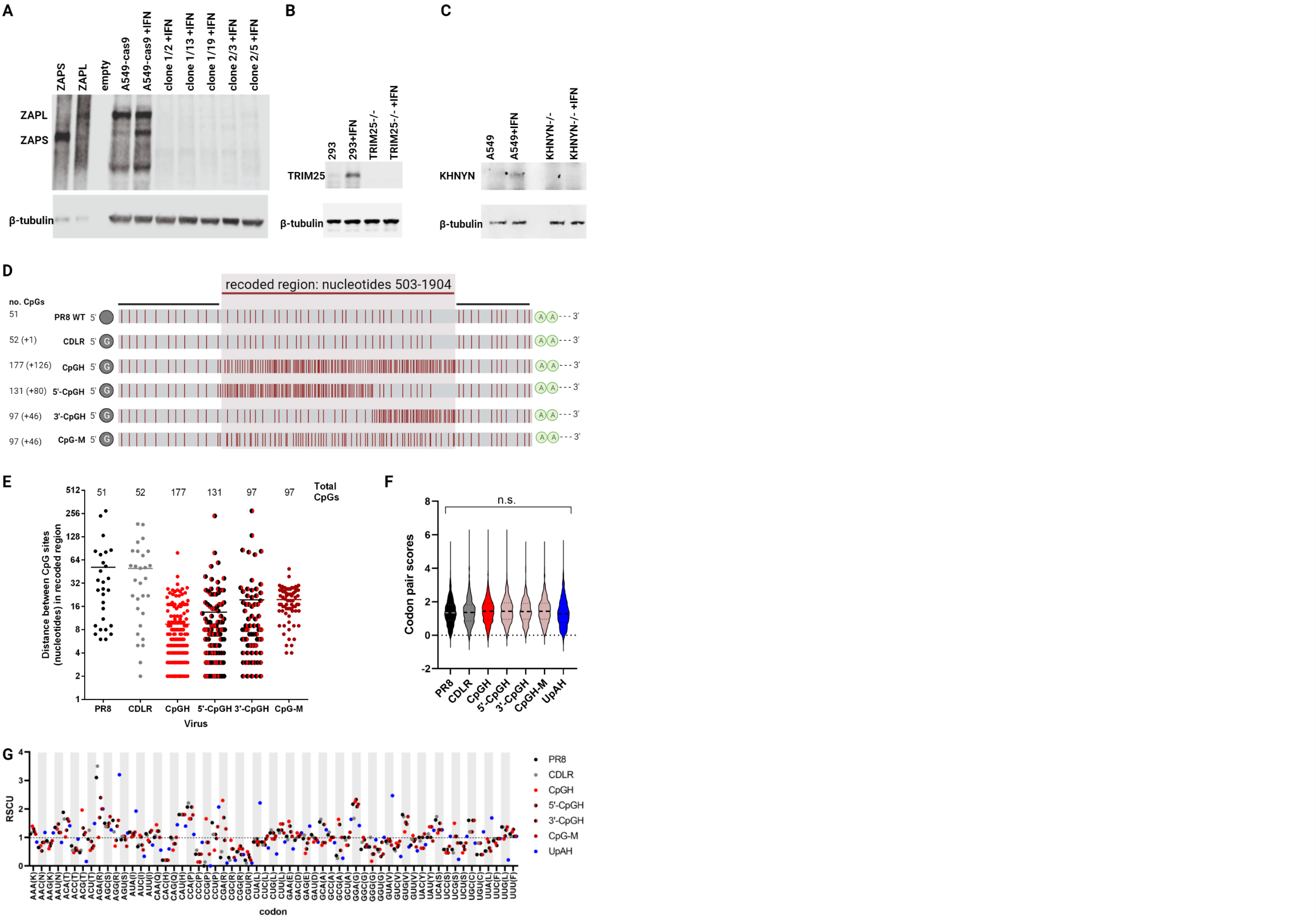

Supplement: S1 Fig — A. ZAP -/- A549 cells (37) were validated by western blotting. B. TRIM25 -/- 293 cells (38) were validated by western blotting. C. KHNYN -/- A549 cells (18) were validated by western blotting. D. Distribution of CpGs in segment 1 of recoded viruses. E. Distance between CpG sites in recoded region of segment 1. F. Codon pair scores across the virus mutant panel. G. Relative synonymous codon usage (RSCU) across the virus mutant panel. (TIF) [file ppat.1011357.s003.tif]

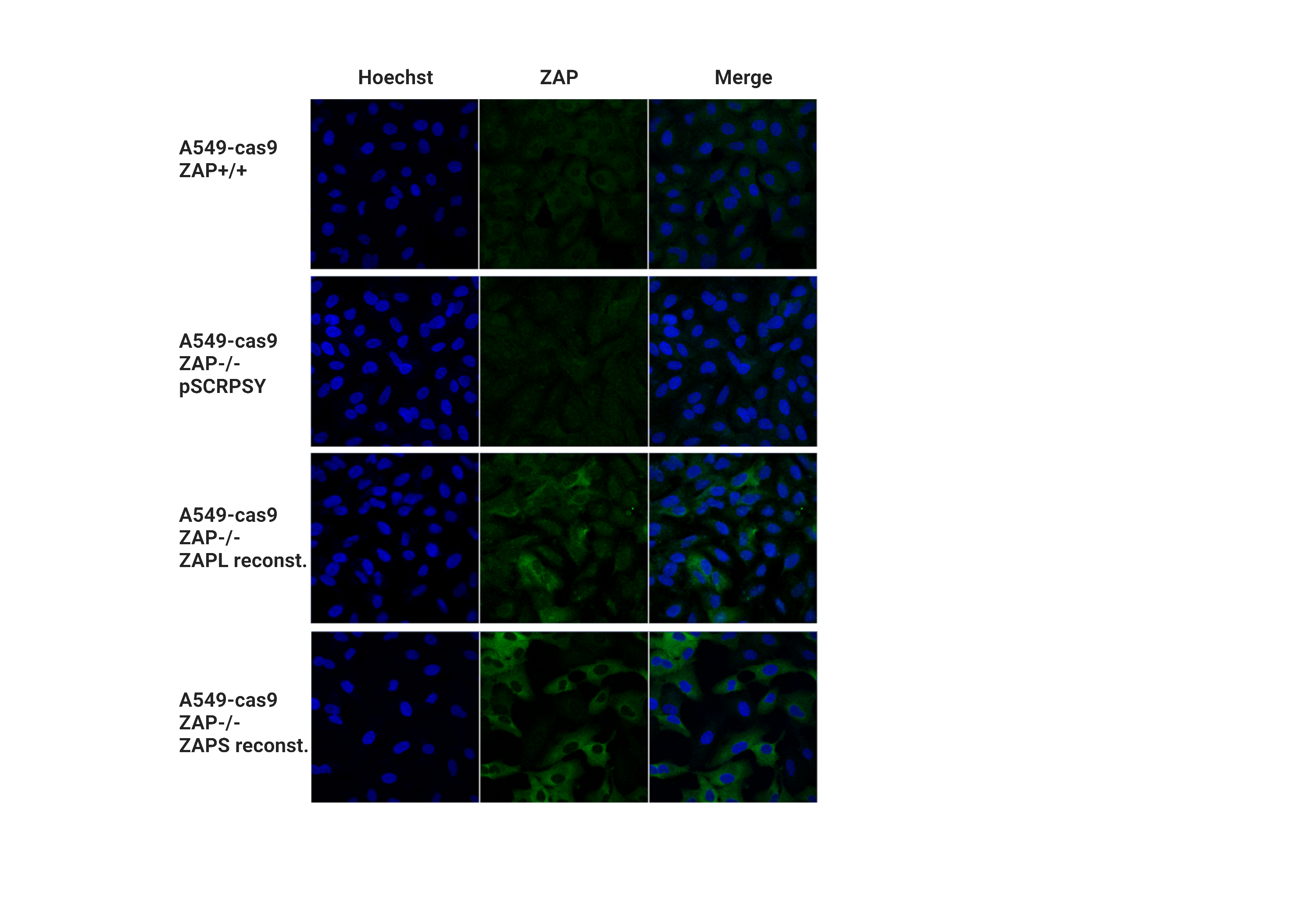

Supplement: S2 Fig — (TIF) [file ppat.1011357.s004.tif]

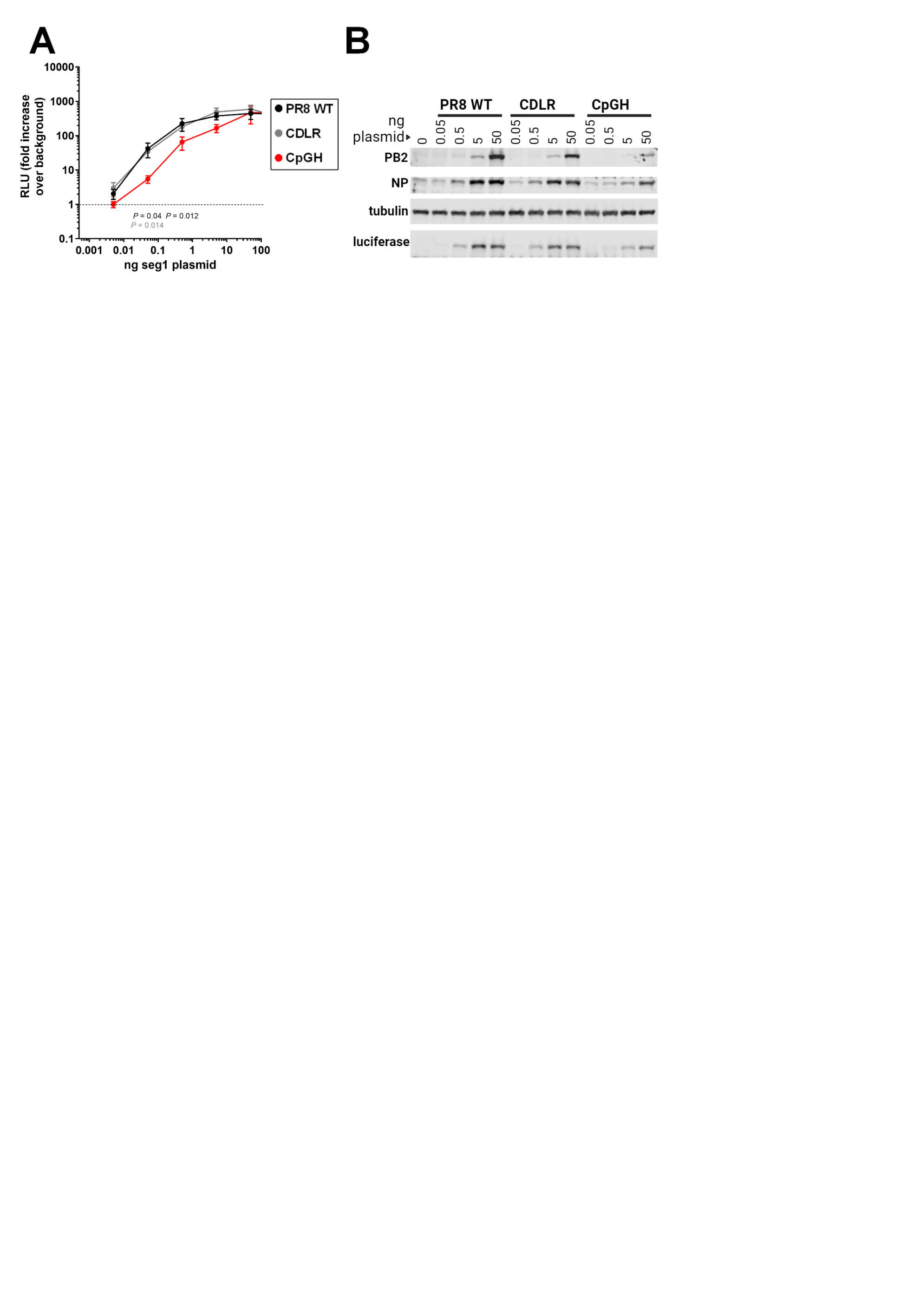

Supplement: S4 Fig — Minireplicon assays that reconstitute the viral polymerase were performed using a luciferase reporter (A, luciferase reporter signal; B, protein signal by western blot). (TIF) [file ppat.1011357.s006.tif]

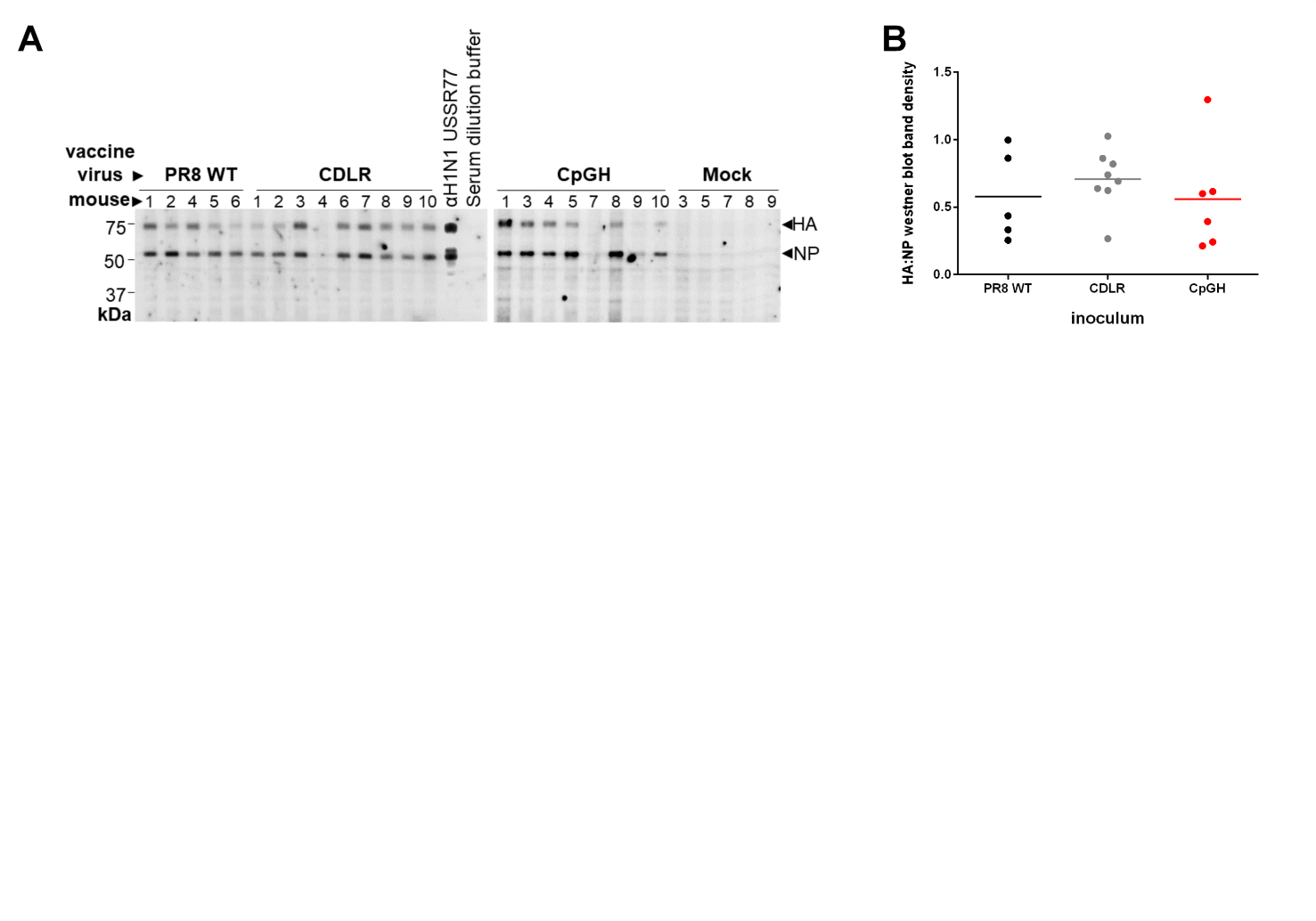

Supplement: S5 Fig — 6-week old female BALB/c mice were infected with wildtype PR8 (n = 6), CDLR (n = 10) or CpGH (n = 10) viruses. After 20 days, tail bleeds were performed and sera harvested from all mice. A. For mice yielding sufficient sera, serum was diluted 1:100 in 2% BSA/ PBS and cross-reacted with cell lysate from MDCK cells infected with wildtype PR8 virus at MOI of 10 for 8 hours. Whole anti-IAV antibody (αH1N1 USSR77) was used as a positive control. Variable cross-reactivity to HA and NP proteins was observed for the different virus inocula, but this variability was consistent across viruses (B). (TIF) [file ppat.1011357.s007.tif]

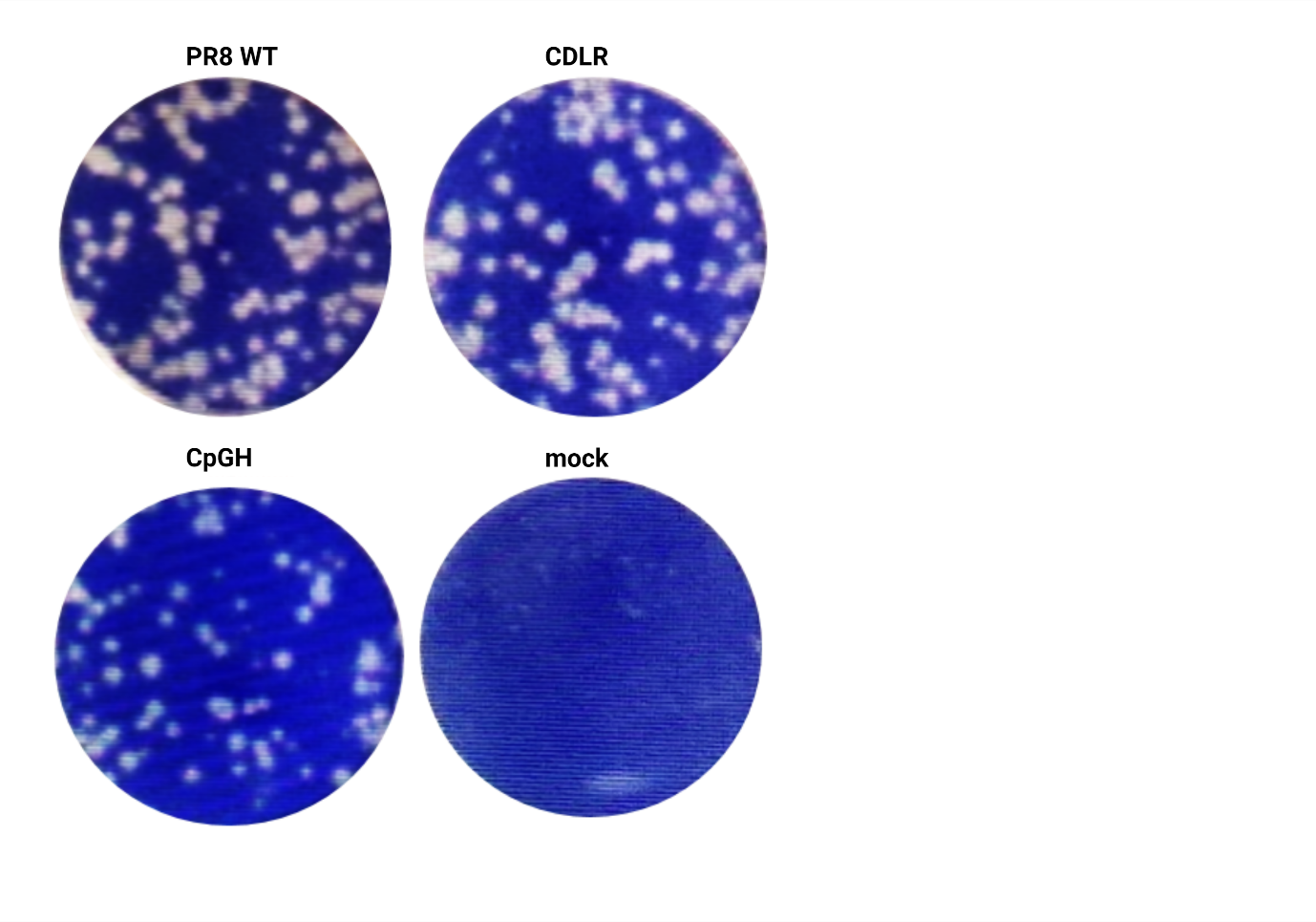

Supplement: S6 Fig — WT PR8, CDLR and CpGH viruses were rescued in embryonated hens’ eggs and titred by plaque assay in MDCK cells. Representative images taken from plaque assays performed at the same time. (TIF) [file ppat.1011357.s008.tif]

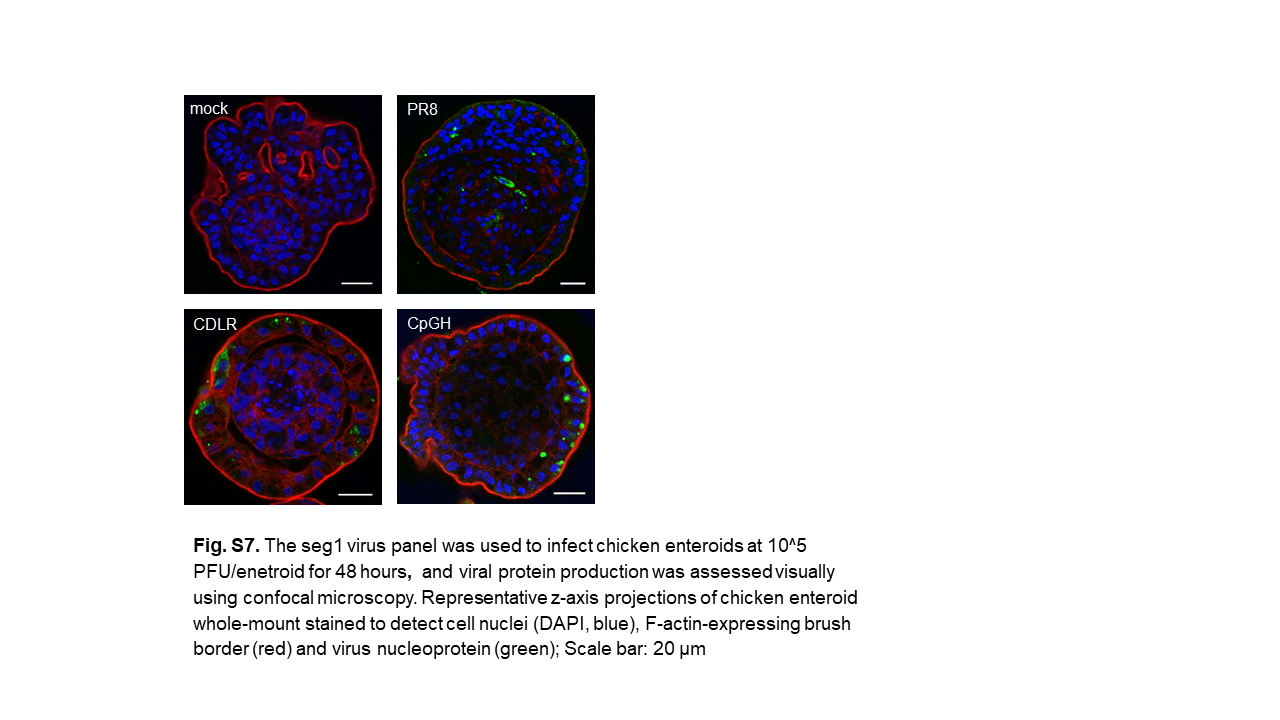

Supplement: S8 Fig — WT PR8, CDLR and CpGH viruses were used to infect chicken enteroids at 105 PFU/enteroid for 48 hours, and viral protein production was assessed visually using confocal microscopy. Representative z-axis projections of chicken enteroid whole-mount stained to detect cell nuclei (Hoechst, blue), F-actin-expressing brush border (red) and virus nucleoprotein (green). Scale bar: 20 μm. (TIF) [file ppat.1011357.s010.tif]

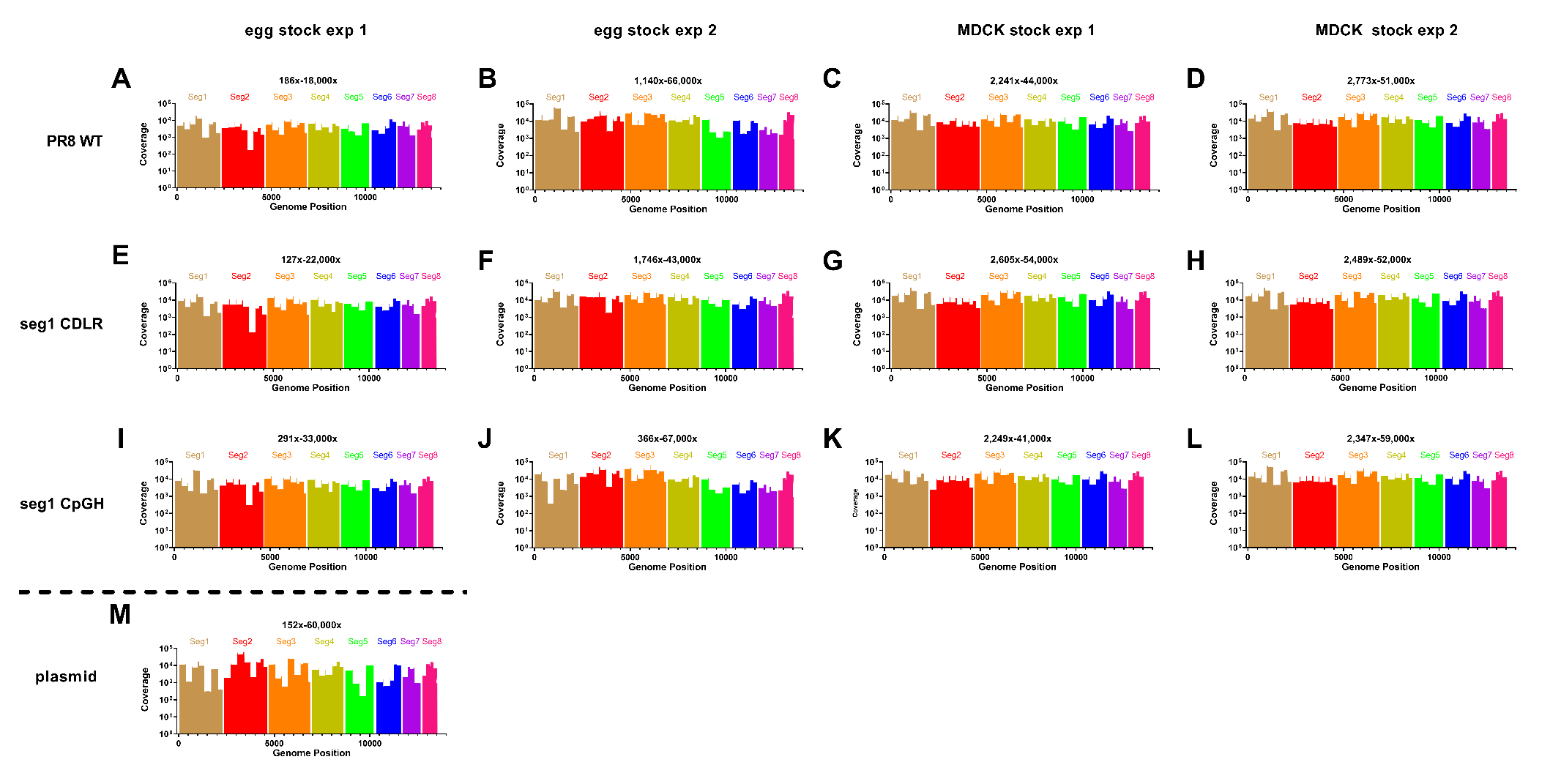

Supplement: S10 Fig — A-D, PR8 wildtype virus; E-H, CDLR; I-L, CpGH; M, input plasmid. (TIF) [file ppat.1011357.s012.tif]

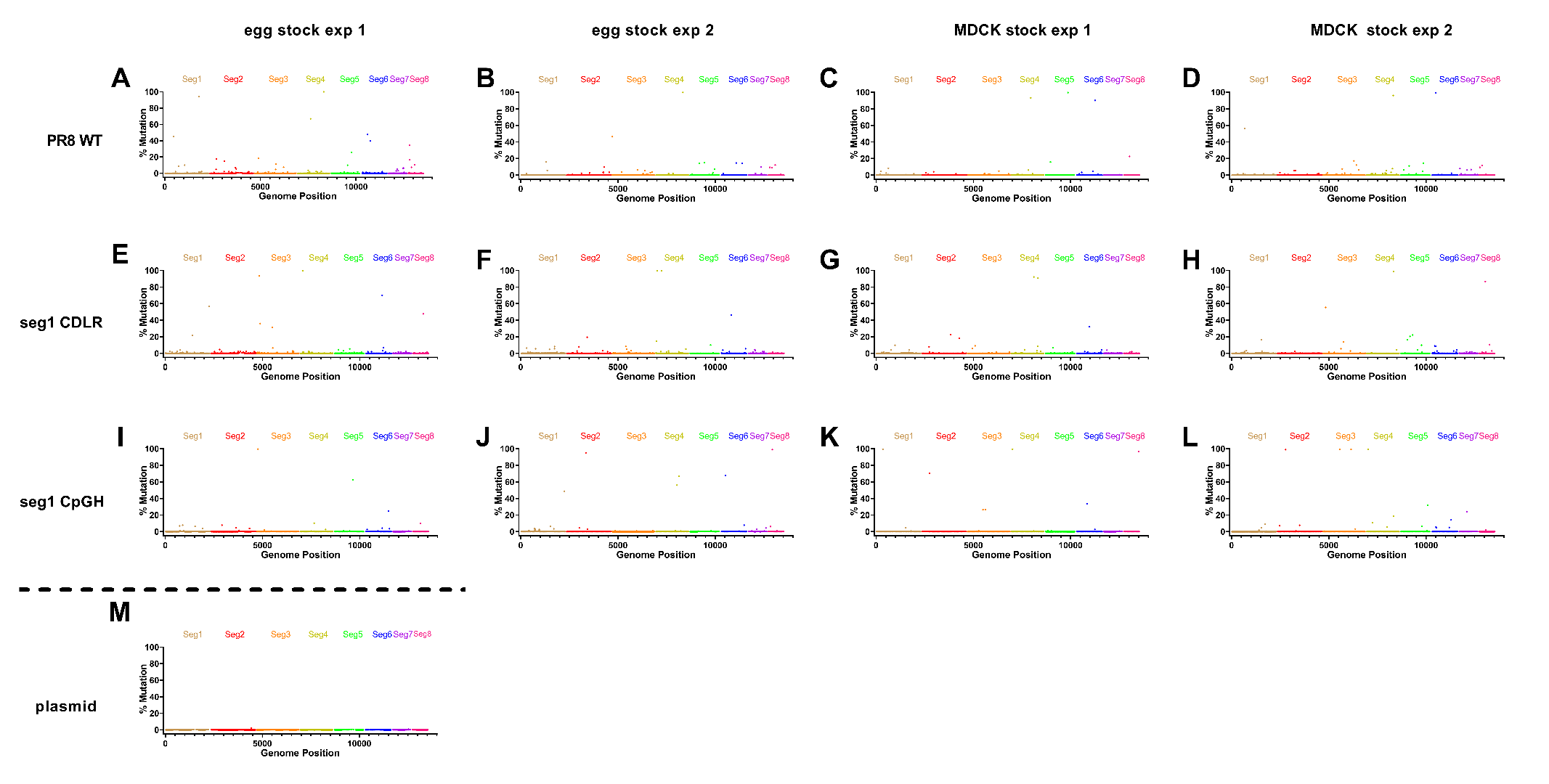

Supplement: S11 Fig — A-D, PR8 wildtype virus; E-H, CDLR; I-L, CpGH; M, input plasmid. (TIF) [file ppat.1011357.s013.tif]
