# Supplementary material for: CpG dinucleotide enrichment in the influenza A virus genome as a live attenuated vaccine development strategy
Source: PLoS Pathog. 2023 May 5;19(5):e1011357. doi: 10.1371/journal.ppat.1011357 (PMC10191365; doi:10.1371/journal.ppat.1011357)
Supplement: Attachment — Submitted filename: Reviewer Response CpG IAV PPATHOGENS-D-22-01903.pdf [file ppat.1011357.s014.pdf]

The Roslin Institute
The University of Edinburgh
Easter Bush Campus
Midlothian
EH25 9RG
27 February 2023

Dear Editors and Reviewers,

Re: PPATHOGENS-D-22-01903

On behalf of all co-authors, please find below response to reviewer comments in blue.

Yours sincerely

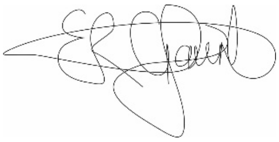

Eleanor Gaunt, corresponding author

Your manuscript was sent out for review and while the work was viewed as a significant addition to the field there were concerns raised by the reviewers that need to be addressed. In particular reviewer #2 raised several important points that need to be addressed to interpret and/or validate the experiments. First of all a description of the statistical analysis utilized throughout the manuscript and an explanation as to the number of times an experiment was performed needs to be included in order to interpret much of the data. In addition, although the animal experiments appear to have 6-10 mice per group, as written, it appears these experiments were only an N of 1. These studies need to be repeated at least 1 time, possibly more depending upon the results and statistical analysis. In addition, reviewer 2 raises the need for additional control viruses as outlined in his review. Both reviewers have also identified areas that need additional discussion of previously published work or findings from your work that warrant further discussion.

We are grateful to both reviewers for their insightful comments, and for their time taken in doing this. We believe that responding to these comments has significantly improved the quality of our manuscript.

Reviewer's Responses to Questions

Part I - Summary
Please use this section to discuss strengths/weaknesses of study, novelty/significance, general execution and scholarship.

Reviewer #1: This paper reports the construction and properties of a variant PR8 influenza A virus with elevated CpG in segment 1. This is a nice extension of recent similar work from the Bieniasz lab to study the effects of CpG addition to virus properties in another virus context (e.g. PMID: 36075961, and less importantly, PMID: 33402534). As has recently been shown with EV-71, viruses can be attenuated in ZAP-positive cells and not in ZAP KO cells. Analysis of viral RNA in CpG-high IFA virus here showed a reduction in segment 1 RNA levels, and also other segments, in ZAP-positive cells. The CpGhigh virus at low dose inoculation was able to induce antibodies in mice, and protect from subsequent challenge with wt virus, while only causing mild symptoms.
The work here shows also that the virus can be propagated to high titer in chicken eggs and cell lines, which are apparently not expressing significant ZAP function, and thus is suitable for large-scale vaccine preparation. Passage of the CpGhigh virus in restrictive A549 cells did not readily result in reversion or loss of the CpG mutations.

I consider this paper to be a significant addition to the literature describing attempts to generate attenuated viruses for vaccine utilization. It is clearly only an extension of the ground-breaking paper from the Bieniasz lab on similar work with EV71, but this is a very important setting (influenza virus). All the work seems solid. The impact of the heavy mutagenesis on replication is perhaps surprisingly small (30x or so in Zap positive cells—and there is a lot of noise) but apparently sufficient to render the virus poorly able to cause symptoms on its own while still inducing protective immunity.
I think a limitation of this whole approach – the dependence of the immunized population on variable ZAP functionality – and the potential for patient variability in their levels of ZAP expression and functionality – needs to be discussed. Essential!

We fully agree the importance of highlighting the limitations of this approach, and in lines 413-15 of the Discussion have suggested the need to integrate multiple attenuation mechanisms. In accordance with this comment, we have therefore added here the sentence ‘Furthermore, a key limitation of this approach is the possibility that an immunized population may vary in ZAP expression levels or functionality’ (lines 411-12).

Reviewer #2: Sharp and colleagues increase the number of CpG dinucleotides in segment 1 of influenza A virus strain Puerto Rico (PR8). They record reduced virus titers of the recoded virus (CPGH) on A549 cells and, to a lower extent, on 293 cells. Using ZAP-negative A549 and TRIM25-negative 293 cells, and using siRNA treatment the authors conclude that reduced virus replication is mediated via the ZAP-TRIM25 axis. In contrast, CpG-rich IAV replicate similar to wild-type viruses or a recoded control virus with similar efficiency on MDCK cells and in embryonated eggs. Further, the authors show that packaging of viral RNAs is not affected by CpG enrichment and that the methylation profile remains unaffected.
The authors claim that the CPGH mutant virus is a promising candidate for generation of an attenuated live vaccine because the virus induces protective immunity in a mouse model of infection and the virus exhibits genetic stability after serial passage in cultured cells.
The strengths of the manuscript are that the experimental procedures were performed and are presented in

a logical and straightforward fashion. Weaknesses include that similar experiments (i.e., an increase of CpG dinucleotides) were done and reported before. Quite a few of the conclusions are hard to follow as details are missing (see below).

Part II – Major Issues: Key Experiments Required for Acceptance
Please use this section to detail the key new experiments or modifications of existing experiments that should be absolutely required to validate study conclusions.

Generally, there should be no more than 3 such required experiments or major modifications for a "Major Revision" recommendation. If more than 3 experiments are necessary to validate the study conclusions, then you are encouraged to recommend "Reject".

Reviewer #1: I have no major issues requiring additional experimentation. There are some writing issues. The review of the history and background of ZAP is in a few places slightly off base.
Lines 48 ff: Actually there are even more spliced forms (see MacDonald papers, PMID: 31118263). Might as well mention this. Probably all the forms (including S and L) bind CpG, since this is due to the N-terminal finger region shared by them all. Doesn't really make sense to suggest that "a consensus mechanism for CpG sensing by ZAP remains unresolved". The structure of the RNA-protein complex is well-established and has been reported (eg. PMID: 31719195).

We have amended the paragraph to add the sentence 'Four ZAP isoforms are currently identified, including two major isoforms, ZAPS (short) and ZAPL (long) and are produced by alternative splicing', with the indicated reference included. We have replaced 'CpG-binding' with 'CpG-mediated'. We have removed the sentence 'Therefore, a consensus mechanism for CpG sensing by ZAP remains unresolved', and cited Meagher et al., as suggested.

The IFN inducibility of ZAPS, and the constitutive expression of ZAPL, and their relative activity has been an issue in the field but the data in Supplementary Fig 1 are striking and very clear and significantly add to the literature. It should be noted that this is in a particular cell line.

To emphasise this, we have replaced 'this system' with 'A549 cells' (line 261, Results), and added ', in the A549 cells used for infections' to line 424 (Discussion).

Lines 113 ff: TRIM25 is not actually "required" but stimulates ZAP activity. The findings in NHDF cells may be due to incomplete KD as suggested, but may also simply be due to lesser need for TRIM25 in these cells. The authors should cite more comprehensive screens for cofactors, which have revealed numerous stimulatory factors (as many as 31 (PMID: 22615998)).

In line 121, we have added 'optimal' to now state that 'TRIM25 is required for optimal ZAP antiviral activity'. We have also added 'or that ZAP-TRIM25 interactions are less important in these NHDF cells' to line 131.The suggested reference identifies ISGs that are co-regulated by ZAP, but TRIM25 does not appear in the suggested reference. Nevertheless, TRIM25 is an ISG. Therefore, the reference highlighted by the reviewer has instead been added to line 59, where the ISG cascade is described. We have also added reference 14, which links to an ISG database ((http://isg.data.cvr.ac.uk/), to line 55.

14. Shaw et al., PLos Biol., 2017. Fundamental properties of the mammalian innate immune system revealed by multi-species comparison of type I interferon responses.

Line 145: The increase in replication of all viruses, including CpG-high ones, with ZAPL expression is curious and needs some discussion. And the defect in replication of all viruses with ZAPS expression, is also odd, though at least the CpG-high virus was more affected than the CpG low virus. To some extent these finding undercut the arguments of the paper.

We agree that this improvement of virus titres in ZAPL-reconstituted cells is interesting, but we do not at this stage have an explanation. We suggest that endogenously expressed ZAPL maintains cell health and consequently is required for optimal virus yield, whereas ZAPS simply undertakes its known antiviral activities. We have added the text 'It is possible that global increases in viral replication are attributable to the

importance of ZAPL for maintenance of cell viability under stress conditions, such as viral infection' with a supporting reference (44) to lines 157-159. ZAPS is a known ISG with antiviral activity and we are therefore unsurprised that its constitutive overexpression impairs replication of the full virus panel (not just the CpGH virus). We have therefore added '…in keeping with its antiviral properties' (lines 160-161).

44. Ly, P.T., Xu, S., Wirawan, M., Luo, D. and Roca, X. (2022) ZAP isoforms regulate unfolded protein response and epithelial-mesenchymal transition. *Proc Natl Acad Sci U S A*, **119**, e2121453119.

Line 182: Wouldn't deamination of C give U, not T, in RNA? The switch to T only occurs in DNA, and only after replication of the deaminated U to A, followed by replication of the A to give T in the next round. I'm thinking this is major faux pas. Correct me if I'm wrong, or fix the text.

We agree that the reviewer is correct and as we do not find any viral genome methylation we have simply removed this paragraph for clarity and brevity.

Line 214: There is a failed logic here. Yes, ZAPS is an ISG (an IFN stimulated gene), but this is not necessarily relevant to its IFN-stimulating activity. These two issues need to be separated and discussed as such.

We apologise for the lack of clarity and have rephrased the text to read' ZAPS has been reported to both stimulate (12) and inhibit (50) type I interferon responses' with appropriate references included (lines 229-30).

12. Hayakawa, S., Shiratori, S., Yamato, H., Kameyama, T., Kitatsuji, C., Kashigi, F., Goto, S., Kameoka, S., Fujikura, D., Yamada, T. *et al.* (2011) ZAPS is a potent stimulator of signaling mediated by the RNA helicase RIG-I during antiviral responses. *Nature immunology*, **12**, 37-44.
50. Schwerk, J., Soveg, F.W., Ryan, A.P., Thomas, K.R., Hatfield, L.D., Ozarkar, S., Forero, A., Kell, A.M., Roby, J.A., So, L. *et al.* (2019) RNA-binding protein isoforms ZAP-S and ZAP-L have distinct antiviral and immune resolution functions. *Nature immunology*, **20**, 1610-1620.

While the number of CpGs are tested (Figure 1F and Supplementary Fig S1D), it has been shown that the spacing and clustering of the CpGs is very important (Bi PMID: 36075961). Some discussion of these issues is needed for the particular design of segment 1 used here, as related to the Bieniasz paper.

We agree the marked differences between the two systems are intriguing, and have considered this in the Discussion lines 416-22. To emphasise the point, we have also added 'This is in stark contrast with a CpG-enriched HIV-1 replicon, in which single CpGs contributed to viral attenuation, and the distance between CpG sites was critical (22). Our CpG-M construct incorporated equivalent CpG density to the HIV-1 replicon but was not significantly attenuated, highlighting that the importance of CpG number and spacing vary between systems' to lines 148-51, along with the reference.

This is a really interesting apparent contradiction. We also note that ZAPL has been reported to bind the 3'UTR of host cell TRAILR4 transcripts, and the 600 nucleotide region that this has been narrowed down to only includes nine CpGs (by our calculations). We speculate that as ZAPS must recognise CpGs in single stranded RNA, and because TRIM25 has undefined RNA binding motif/ structure, RNA structure is a key determinant of ZAPS-sensitivity. Others have reported an increased ZAPS-sensitivity if adenosine enrichment is also included around CpG sites, and as adenosine is the largest base, this is likely to enhance single-strandedness and consequently ZAPS-sensitivity. However, this is entirely speculative and therefore we do not include this in the paper.

Surely this important paper should be cited?
The role of ZAP and OAS3/RNAseL pathways in the attenuation of an RNA virus with elevated frequencies of CpG and UpA dinucleotides. Odon V, Fros JJ, Goonawardane N, Dietrich I, Ibrahim A, Alshaikhahmed K, Nguyen D, Simmonds P. Nucleic Acids Res. 2019 Sep 5;47(15):8061-8083. doi: 10.1093/nar/gkz581. PMID: 31276592

Absolutely! We have now cited this paper in line 120 (reference 41), where the differences between our findings and the Odon paper are now described, and in the discussion (lines 386 and 435).

Also:
CpG-Recoding in Zika Virus Genome Causes Host-Age-Dependent Attenuation of Infection With Protection Against Lethal Heterologous Challenge in Mice.

Trus I, Udenze D, Berube N, Wheler C, Martel MJ, Gerdts V, Karniychuk U. Front Immunol. 2020 Jan 24;10:3077. doi: 10.3389/fimmu.2019.03077. eCollection 2019. PMID: 32038625

We have now cited this paper in the discussion (ref. 65, lines 386 and 435).

Reviewer #2: 1) Nowhere in the manuscript is any reference to the statistical analyses that were conducted to be found. Hence, this reviewer is unable to accept or reject many of the conclusions made herein.

We apologise for this omission.
We have added a section to the methods to describe the statistical analyses undertaken: '**Statistical analyses and data presentation.** For all *in vitro* experiments, at least 3 biological repeats with 3 technical repeats were performed. Individual data points represent independent biological repeats, calculated from a mean of two or three technical repeats, and horizontal bars represent the mean. To assess for statistical differences between multiple groups, one way ANOVAs were performed for multiple comparisons. For comparisons across two groups, ratio paired t tests were performed in Graphpad Prism v9 unless data are presented as ratios, in which case paired t tests were performed.' (lines 787-92).

2) The scrambled/control virus CDLR where "codon rearrangement" was performed needs to a) be explained better and b) complemented by viruses in which, for example, UpA's are enriched and/or codon/bicodon frequencies are altered.

a) We apologise for the lack of clarity, and have expanded the text in the Methods section to describe the CDLR control in more detail: 'A control mutant, 'seg1-CDLR', was also designed which was synonymously recoded over the same region, by reordering degenerate codons (e.g., the CUU and CUC codons for leucine may be switched within the coding sequence). The frequency of CpGs was not altered from the wildtype in this control. Using this approach, nucleotide, UpA dinucleotide and codon frequencies were also maintained. This control will test whether any previously unidentified RNA sequences or structures that are required for optimal virus replication have been unknowingly disrupted within the recoded region' (lines 464-70).
b) We agree that viral attenuation imparted using a method predicted to be ZAP-independent, e.g. UpA enrichment, will further support our data showing that the ZAP-sensitive phenotype observed is specific for CpG recognition. We have therefore designed a UpAH virus with 115 UpA dinucleotides added through synonymous mutations introduced over the same region of segment 1, rescued viruses, and performed quadruplicate (biological repeat) infections in the A549 and paired ZAP-/- cells with the full virus panel. The generated data showed that both CpGH and UpAH viruses were defective in A549 cells, but only the CpGH virus recovered fitness in ZAP-/- cells:

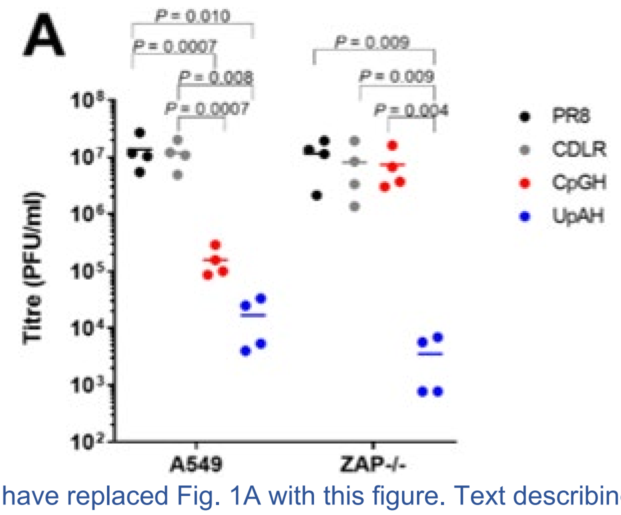

We have replaced Fig. 1A with this figure. Text describing the result has been added (lines 116-20): 'A construct that was designed to be attenuated by the introduction of UpA dinucleotides added across the same region of segment 1 (UpAH) was also defective in A549 cells, but in contrast with the CpGH virus, did not recover fitness in ZAP-depleted cells (**Fig. 1A**). This illustrates the specificity of ZAP for alleviating CpG-mediated attenuation, and is in contrast with paralogous experiments using echovirus-7, where an attenuated UpAH virus had fitness restored when ZAP was depleted'.
The methods have been amended to include 'A further control construct enriched for UpA dinucleotides ('UpAH'), was designed in the same way, with maintained nucleotide, CpG dinucleotide and codon frequencies' (lines 470-72).
Sequence information and sequence FASTA have been added to Tables 1 (p19) and S1 (p49-50) respectively.

3) The animal experiments, apparently, were not done in a randomized, blinded fashion? In addition, since the mice were group-housed as I understand from the methods described, one could argue that the groups have an N=1. As the CpGH virus seemed not to be attenuated until day 3 after inoculation, there should be at least one repeat of the experiment.

We apologise for the brevity in describing the methods for the animal experiments, and have made changes accordingly which indicate that the experiments were undertaken in a blinded fashion. Prior to infection, mice were housed randomly, and mean weights across groups were calculated and found to be not statistically different across groups. The following text has been added to the methods: 'Prior to infection, mice were weighed to ensure that there were no differences in mean weights or deviations across groups' (lines 707-8). We have also added 'Groups were blinded during weighing' (line 711) to reflect the methodologies used.

From our mouse data, we draw two conclusions:

1. The CpGH virus is attenuated *in vivo*

2. Prior infection with attenuated CpGH virus offers protection from challenge with a potentially lethal dose of wildtype virus.

1. The CpGH virus is attenuated *in vivo*. In Fig. 6 from the original manuscript, panels A and C represent independent experiments in which BALB/c mice were challenged with 200 PFU or 20 PFU of the virus panel respectively. Significant attenuation of the CpGH virus was observed in both experiments. Attenuation of the CpGH virus became apparent at different times post infection, reflective of the inoculation dose and/ or the number of mice used in each experiment (panel A = 5 mice, panel C = 10 mice). In the high dose infections (A), the initial 'big hit' of virus is apparent at day 3 with lower weight loss in the CpGH group not attaining statistical significance. However, mice infected with CpGH virus showed signs of recovering quickly than those infected with WT or control virus (which could be because of lower CpGH virus replication and / or the immune responses mounted). In the low dose infections (C), over each round of viral replication the WT virus is replicating more effectively than the CpGH virus, and this becomes significantly apparent by day 7. **We therefore believe that this constitutes two independent experiments, included in the paper, which support our conclusion that the CpGH virus is attenuated *in vivo*.**

2. Prior infection with the CpGH virus offers protection from challenge with a potentially lethal dose of wildtype virus. Figures 6C-G and FigureS5 from the original manuscript, together show that exposure to CpGH virus offers protection from a potentially lethal dose challenge with wildtype virus. Ongoing work in the lab aims to test the limits of the system, and we have supporting data from an independent experiment that recapitulates this result (shown below; not presented in the manuscript). To test the limits of protective dose, 5 BALB/c mice were inoculated with 1 PFU of CpGH virus (i.e., at the limit required to establish infection), and then were challenged with 200 PFU PR8 WT virus 21 days later. Consistent with a dose at the limit of infection, antibody responses to the initial infection with CpGH virus were detected in 2/5 mice, indicating that 2 mice were successfully infected, whereas 3 were not; weight loss and clinical signs were not observed in any mice (below, panel A). However, when mice were challenged with WT virus, only the mice with antibody responses were fully protected from challenge. Conversely, mice without antibodies developed infections that caused clinically moderate or severe outcomes (panels B and C):

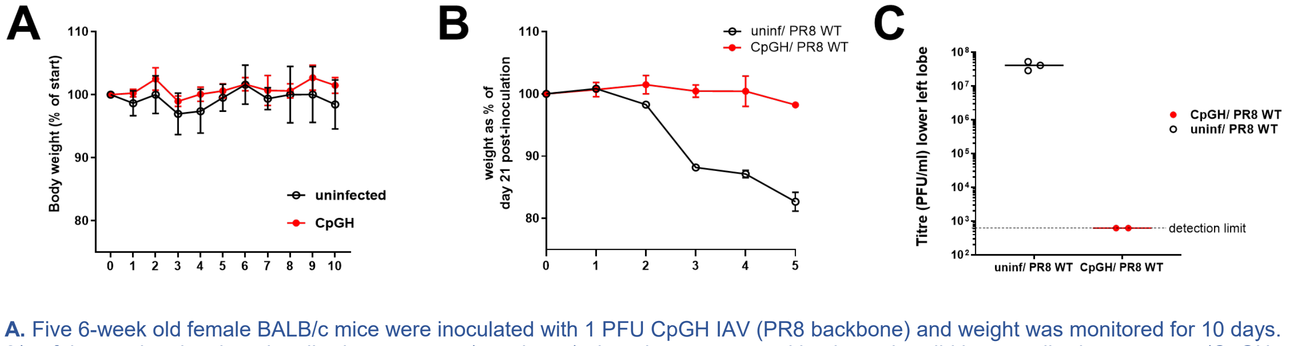

A. Five 6-week old female BALB/c mice were inoculated with 1 PFU CpGH IAV (PR8 backbone) and weight was monitored for 10 days. 2/5 of these mice developed antibody responses (not shown); the mice are grouped by those that did have antibody responses (CpGH, red line and circles) and those that did not (uninfected, black line and open circles). **B.** After 21 days, the same mice were challenged with 200PFU WT PR8 virus, and weight was monitored for 5 days. **C.** Lower left lung lobes were harvested and virus was titred.

We are therefore fully confident that sub-clinical infection with CpGH virus delivers protection from challenge with a potentially lethal dose of WT virus. The data presented here are part of ongoing work better suited to asking a different question, and are therefore not included in the manuscript.

Given the above, we are convinced of the biological reproducibility and scientific robustness of the data presented in the paper (otherwise it would not be included) and that further repetition of any animal experiments, especially under the high severity conditions of the experiment in Fig. 6A, would be unnecessary use of animals. The UK Home Office guidance on operation of ASPA states that measures to avoid duplication of studies including review of data must be taken. Viewing the data from multiple animals in multiple independent studies as a whole, all conclusions in this paper are robustly supported. As we have advanced the experimental design beyond the data presented in panel A to successful challenge studies, performing a regressive and high severity experiment on 21 mice is therefore contrary to Home Office regulations and potentially an ethical violation of our Project and Personal licenses. Full guidance is available: https://assets.publishing.service.gov.uk/government/uploads/system/uploads/attachment_data/file/662364/Guidance_on_the_Operation_of_ASPA.pdf.

The infections in panel A were done at 200 PFU – a potentially lethal (and therefore very high) dose, not reflective of a vaccine dose. Nevertheless, we agree with the Reviewer in that this may be important in immunocompromised recipients. We have therefore added to the Discussion 'When mice were infected with a high dose of CpGH virus (Fig. 6A), the initial response to infection was not dissimilar to that seen during WT virus infection. While the dose used was high, this highlights the importance of considering how CpGH-attenuated viruses may replicate in immumocompromised hosts' (Lines 408-11), where we also discuss the possibility of multiple layers of attenuation. Our ongoing studies investigating how these viruses replicate in IFNAR-/- mice are more pertinent to this important point, but are beyond the scope of this study.

Part III – Minor Issues: Editorial and Data Presentation Modifications
Please use this section for editorial suggestions as well as relatively minor modifications of existing data that would enhance clarity.

Reviewer #1: See above.

Reviewer #2: 1) Line 108 and Figure 1: The term "Low MOI" has to be clarified (.1, .01?). I also assume that the dots represent independent replicates and that the horizontal bar mean/median? Why were foreskin fibroblasts used?
There is reference to the "significant" reduction in virus titers when wt/CDLR is compared to CpGH. However, there is substantial variability (~20-fold) within wt. Hence, growth kinetics rather than a single time point (why 48 h) would seem appropriate.

We apologise for the lack of clarity regarding MOI and have edited the methods section (lines 521-25): 'For single cycle infections using a high multiplicity of infection (MOI), an MOI of 10 for 24 hours was used. For low multiplicity multi-cycle infections, an MOI of 0.01 was used in A549, 293 and MDCK cells. For NHDF infections, an MOI of 3 was used for 24 hour infections due to the lower permissiveness of these cells'.

We have added 'Individual datapoints represent independent biological repeats, calculated from a mean of two or three technical repeats, and horizontal bars represent the mean' to the methods, lines 788-90.

Foreskin fibroblasts were used as these cells represented an opportunity for us to test our virus panel in primary cells known to be permissive for IAV infection, and in which the experimental conditions for ZAP and TRIM25 knockdown in these cells had previously been established (reference 43; Lin *et al*). siRNA knockdowns were also trialled in A549 cells, but the knockdown was less efficient and less consistent.

Re: growth curves: The purpose of Fig. 1 is to show that CpGH virus is defective in ZAP-competent cells, but the defect is abrogated/ reduced when ZAP co-factors are removed or depleted. We tested various conditions to illustrate this clearly and simply, including various MOIs and timepoints. We found endpoint titres at 48 hours post-infection with low MOI input yielded the most consistent data, which is why we chose to present this. At 24 hours, we often found that the CpGH virus was replicating poorly to the point that we

did not get enough plaques to yield reliable counts, and the data were noisy, probably due to stochastic variability in the number of cells initially infected and viral growth being in exponential phase. At 72h, extensive cell death corresponded with drop offs in virus titres, likely due to the release of proteases harmful to virion integrity. Presenting additional, noisy, data would not change the conclusions of this figure but will diminish its clarity and quality.

We acknowledge that absolute titres can deliver inherent variability between replicates, but the magnitude of replication defect seen in our CpGH virus was extremely consistent. We deliberately chose to present the raw titres rather than normalised fold differences in virus titres so maximise the transparency of our findings (for example, we saw significant variation in viral titres in different cell lines, which may be important when we consider how ZAP affects replication dynamics).

2) Lines 178 -218 and Figure 3: How do you explain that methylation patterns of the CpGH virus remain unaltered? was that the case for the other CpG mutant viruses (5', 3') as well? If there is no difference, is there a need to show the data?

In DNA, cytosines in the CpG conformation are frequently methylated as a mechanism to regulate transcription. To date, cytosine methylation in RNA (cellular or viral) has not been associated with any specific motif. There are several examples of base modifications that occur in both DNA and RNA (via discrete mechanisms), and so we considered the possibility that by increasing CpG content, we may alter cytosine methylation patterns of viral RNA. We found that the wildtype PR8 virus genome has no cytosine methylation sites. We feel that it is important to include these data as an absence of cytosine methylation in the IAV genome (Fig. S3) is novel, and adds to the growing body of literature on post-transcriptional modifications (or their absence) in viral genomes.

Similarly, we found no evidence of methylation in the CpGH virus genome. Over years of presenting our research to colleagues including virologists, RNA biologists, and geneticists, we have been asked repeatedly whether we may have altered methylation patterns by adding CpGs. It is important to demonstrate that addition of CpGs is not introducing aberrant methylation sites, and as far as we are aware it has not previously been specifically investigated whether CpGs in RNA are prone to methylation.

As all data were negative, we see no reason to pursue investigations for the constructs with intermediate CpG profiles (5’, 3’, M).

3) Figure 4: Again, the differences in virus titers are quite variable between replicates. In addition, the conclusion that N and P2 protein levels are down in CpGH viruses is not supported by the data as presented (Fig. 4C).

For these experiments we used high MOI infections with the 24h timepoint harvested, and so possibly some cell death is contributing to the variability between replicates. We didn’t normalise data because we didn’t want to hide or exclude outlying replicates. Mycoplasma testing and consistent batches of FBS were used throughout all experiments. The differences we are examining are robustly supported statistically.

We stand by our assessment that NP and PB2 levels are lower for CpGH viruses (Fig. 4C), although we acknowledge that variation in tubulin levels across lanes preclude the immediate apparentness of this. Below we present the band densities normalised to tubulin (quantified in Image J), where it can clearly be seen that CpGH viral proteins are present at lower levels when compared to PR8 and CDLR (red arrows draw attention to lanes representing CpGH constructs). We have added ‘quantification of band densities normalised to tubulin not shown’ (lines 252-3).

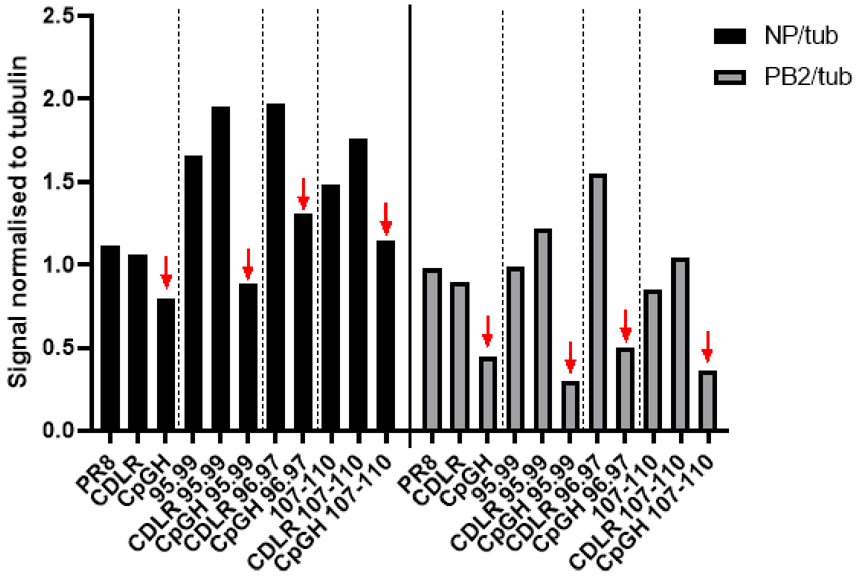

4) Figure 5: PB2 and N seem to be expressed at lower levels in the case of the CDLR virus (Fig. 5C). Why? Also, there is lower levels of vRNA but not =RNA in the case of CDLR. Can you explain?

We selected one blot to present from 3 repeats of this timecourse. However, PB2 and NP bands were consistent for PB2 and NP in the other two blots, and so we have replaced the blot with that from a different timecourse to show this. Due to the lack of viral protein detection at 8 hpi in the new blot, we have removed the 6 hpi timepoint.

Re lower vRNA for CDLR – this subtle difference was not reproducibly observed, and so we have repeated this experiment and replaced this image. The segment 5 vRNA and mRNA probes were more sensitive than the segment 1 probes (as can be seen in the IVT controls from the previous image). While segment 1 and segment 5 were probed for on the same membrane, we have now split these into different exposure intensities so that the control IVT bands are of equivalent densities. We believe that this gives a much clearer indication of CpGH band density relative to the controls. We have added to the figure legend 'Segment 1 and segment 5 vRNAs or +RNAs were probed for on the same membrane, but due to differences in sensitivities of the probes, different exposures are presented for the two segments.'

In addressing this comment we realised an omission from the methods, and so have added in details of how the 18s loading control image was obtained (lines 691-2: 'Prior to transfer, gels were and scanned using a LICOR Odyssey Fc imaging system to visualise 18s rRNA.').

5) Figure 6 and Lines 280-306/667-680: The animal experiments needs a better design/repeat and proper statistical evaluation (blinded investigators, randomization, exclusion of cage effect). Also, how do you explain the parallel curves of all three viruses until day 3 (Fig. 6A) and the recovery of the CpGH mice? How would the CpGH virus do in immunosuppressed mice, which would be important to know if this virus were to be considered as a live attenuated vaccine?

This has mainly been discussed above, with the exception of immunocompromised individuals. We have added consideration of polymorphisms in ZAP gene across a vaccinated population in line with comment from Reviewer 1. We fully agree that what happens in immunocompromised mice is a very important question; however, our ongoing studies investigating how these viruses replicate in IFNAR-/- mice are beyond the scope of this study.

6) Figure 7: Again, on MDCK, titers stretch over two orders of magnitude. Again, growth kinetics would seem more appropriate than just one time point.

We apologise for lack of clarity - the data presented are virus stock titres, and are intended to reflect how much virus can be obtained using the methods applied during vaccine propagation. The associated text has been edited to make this clearer (line 332 now reads '…titres from virus rescued in MDCK cells were

equivalent across the virus panel’). During virus rescue, viruses generated from transfected HEK293T cells is passaged onto MDCK cells or into eggs without prior titration, and therefore there will be variability in the amount of inoculum used into the virus propagation systems. This is far more apparent in MDCKs than in eggs.
