# Supplementary material for: CpG dinucleotide enrichment in the influenza A virus genome as a live attenuated vaccine development strategy
Source: PLoS Pathog. 2023 May 5;19(5):e1011357. doi: 10.1371/journal.ppat.1011357 (PMC10191365; doi:10.1371/journal.ppat.1011357)
Supplement: Attachment — Submitted filename: Reviewer Response CpG IAV PLoS Path 4Apr23.docx [file ppat.1011357.s015.docx]

The Roslin Institute

The University of Edinburgh

Easter Bush Campus

Midlothian

EH25 9RG

3rd April 2023

Dear Editors,

**Re: PPATHOGENS-D-22-01903**

On behalf of all co-authors, please find below response to reviewer comments in blue.

Yours sincerely

Eleanor Gaunt, corresponding author

The authors both agree that this is an important piece of work and that the majority of the concerns raised during the first review have been addressed adequately by the authors. However, reviewer #1 raises an important point regarding the need to place this work in the context of published literature in the field, in particular to calculate the codon and bicodon usage/scores and to discuss the findings of your work in the context of what exists in the literature and the contradictions/controversy that exists.

We thank the editors for this clear summary. Accordingly, we have added codon/ bicodon usage and expanded placing our work in context in the field as described in the reviewer responses below.

Reviewer Comments (if any, and for reference):

Reviewer's Responses to Questions

**Part I - Summary**
Please use this section to discuss strengths/weaknesses of study, novelty/significance, general execution and scholarship.

Reviewer #2: The authors have addressed most of my comments. Importantly, the authors have included the description of the statistical analyses and one of the mutant viruses (UpAH), which behaved as predicted: It has a clear growth defect, which, in contrast to the CpGH virus, cannot be rescued on ZAP-/- cells. However, this result obviously raises the question what the mechanism of reduced growth of this virus is.
The authors raise the issue of codon usage, which was unaltered in the recoded viruses. However, given the controversies between the "Simmonds" and the "Wimmer/Skiena/Mueller/Coleman" groups on the reason for recoding, it would seem necessary to a) calculate not only codon but also bicodon usage/scores and b) at least discuss the findings in the context of the literature abound on recoding of flu viruses. There is not a single reference referring to this earlier work. Here is a (certainly not comprehensive) selection of relevant literature:

1: Groenke N, Trimpert J, Merz S, Conradie AM, Wyler E, Zhang H, Hazapis OG,
Rausch S, Landthaler M, Osterrieder N, Kunec D. Mechanism of Virus Attenuation
by Codon Pair Deoptimization. Cell Rep. 2020 Apr 28;31(4):107586. doi:
10.1016/j.celrep.2020.107586. PMID: 32348767.

2: Kaplan BS, Souza CK, Gauger PC, Stauft CB, Robert Coleman J, Mueller S,
Vincent AL. Vaccination of pigs with a codon-pair bias de-optimized live
attenuated influenza vaccine protects from homologous challenge. Vaccine. 2018
Feb 14;36(8):1101-1107. doi: 10.1016/j.vaccine.2018.01.027. PMID: 29366707.

3: Broadbent AJ, Santos CP, Anafu A, Wimmer E, Mueller S, Subbarao K. Evaluation
of the attenuation, immunogenicity, and efficacy of a live virus vaccine
generated by codon-pair bias de-optimization of the 2009 pandemic H1N1 influenza
virus, in ferrets. Vaccine. 2016 Jan 20;34(4):563-570. doi:
10.1016/j.vaccine.2015.11.054. Epub 2015 Dec 2. PMID: 26655630; PMCID:
PMC4713281.

4: Mueller S, Coleman JR, Papamichail D, Ward CB, Nimnual A, Futcher B, Skiena
S, Wimmer E. Live attenuated influenza virus vaccines by computer-aided rational
design. Nat Biotechnol. 2010 Jul;28(7):723-6. doi: 10.1038/nbt.1636. Epub 2010
Jun 13. PMID: 20543832; PMCID: PMC2902615.

5: Wimmer E, Mueller S, Tumpey TM, Taubenberger JK. Synthetic viruses: a new
opportunity to understand and prevent viral disease. Nat Biotechnol. 2009
Dec;27(12):1163-72. doi: 10.1038/nbt.1593. PMID: 20010599; PMCID: PMC2819212.

Of particular interest seems reference 1, in which high CpG content of a recoded influenza A virus seems not to have caused any growth defect in cultured cells including A549. Is the difference observed here to published data a segment-specific phenomenon? Is it a strain-specific phenomenon? At any rate, I think the results presented here need to be discussed in the light of earlier and at least partially contradictory results.

A) Given that our CpGH virus rescues in ZAP-/- systems, we are fully confident that codon and bicodon usage do not explain the attenuation of our mutants. However, given the history of this field, we agree that it is important to understand how much these parameters vary across our mutants. Bicodon usage data have been added (**Fig S1F**). How well codon usage reflects host is already presented in the CAI column of Table 1 (CAI = codon adaptation index). To expand on this, we have added a figure indicating relative synonymous codon usage across the mutant panel, with stop codons and methionine (AUG only) and tryptophan (UGG only) excluded (**Fig. S1G**). We have added the text ‘Codon pair and codon usage distributions of the virus panel are summarised (**Fig. S1F-G**)’ to line 140, and added ‘Codon pair and codon usage was calculated for the mutant panel using SSE v1.4’ to methods (line 447-8).

B) The papers listed describe investigations of how codon pair bias impacts virus replication, but only the most recent reference, reference 1, accounts for disrupted dinucleotide bias. Therefore, we take an agnostic view that the two mechanisms, CpG sensing and codon pair biases, may be discrete. To this end, we have added the text:

‘Historically, use of synonymous recoding to attenuate IAV replication has focussed on disrupting codon pair biases (71-74). In a more recent study that sought to uncouple the attenuative effects of codon pair bias deoptimisation from CpG enrichment in IAV, Groenke and co-authors found no attenuation of a CpGH IAV (75). However, their CpGH IAV had 45 CpGs added, which was most comparable to our CpG-M mutant with 46 CpGs added and which was also not attenuated (**Fig 1F**) (though their experiments were undertaken using WSN strain recoded in segment 6, encoding the NA surface glycoprotein). Nevertheless, when disrupting codon pair biases, Groenke and colleagues maintained CpG frequencies in their recoded viruses, and found that use of disfavoured codon pairs attenuated IAV replication. Here, we have demonstrated the specificity of CpG-mediated attenuation through the restoration of CpGH virus fitness in ZAP-/- systems. Therefore, CpG enrichment and introduction of disfavoured codon pairs may represent discrete attenuation mechanisms’.

-to lines 387-396 of the Discussion.

In this paragraph, we have cited references 1-4 indicated by the reviewer, but have not cited reference 5 as this is a review that is not specific to influenza.

We also cite Yang et al., PNAS, 2013 ‘Deliberate reduction of hemagglutinin and neuraminidase expression of influenza virus leads to an ultraprotective live vaccine in mice’.

**Part III – Minor Issues: Editorial and Data Presentation Modifications**
Please use this section for editorial suggestions as well as relatively minor modifications of existing data that would enhance clarity.

Reviewer #1: I think the minimal concession about the potential issues of patient variability in ZAP functionality should be extended -- this could be a serious problem in the application of these viruses in vaccines.

We have added the text ‘The clinical importance of mutations in the ZAP-encoding gene, ZC3HAV1, was highlighted in a recent study that linked a mutation in this gene with COVID-19 disease severity (67)’ to lines 382-383, along with a supporting reference, so highlight the real-world implications of ZAP polymorphisms in the human population.
